# Pyrrocidines A and B demonstrate synergistic inhibition of *Fusarium verticillioides* growth

**DOI:** 10.3389/fmicb.2024.1480920

**Published:** 2025-01-09

**Authors:** Lily W. Lofton, Quentin D. Read, Hailey L. Hamilton, Anthony E. Glenn, Jaci A. Hawkins, Trevor R. Mitchell, Scott E. Gold

**Affiliations:** ^1^Department of Plant Pathology, University of Georgia, Athens, GA, United States; ^2^Toxicology and Mycotoxin Research Unit, United States National Poultry Research Center, Agricultural Research Service, United States Department of Agriculture, Athens, GA, United States; ^3^Southeast Area, Agricultural Research Service, United States Department of Agriculture, Raleigh, NC, United States

**Keywords:** *Fusarium verticillioides*, *Sarocladium zeae*, fumonisin, pyrrocidine, maize, biological control, secondary metabolites, fungal–fungal interactions

## Abstract

*Fusarium verticillioides*—a mycotoxigenic fungus and food safety threat—coinhabits maize kernels with *Sarocladium zeae*. This protective endophyte produces secondary metabolites of interest, pyrrocidines A and B, which inhibit the growth of *F. verticillioides* and specifically block fumonisin biosynthesis. Previous transcriptomic analyses found *FvZBD1* (FVEG_00314), a gene adjacent to the fumonisin biosynthetic gene cluster, to be induced over 4,000-fold in response to pyrrocidine challenge. Deletion of *FvZBD1* resulted in dramatic increases in fumonisin production (FB_1_ >30-fold). Here, using pyrrocidine dose-response assays, we discovered a potent synergy between pyrrocidines A and B, where they functioned powerfully together to inhibit *F. verticillioides* growth. Further, results provided evidence that *FvZBD1* confers partial tolerance to pyrrocidines, particularly pyrrocidine A, and that pyrrocidine functions through *FvZBD1* to effectively eliminate fumonisin biosynthesis. Additionally, we showed that the *FvABC3* (FVEG_11089) mutant, earlier described as hypersensitive to pyrrocidine, is particularly sensitive to pyrrocidine B. Thus, pyrrocidine A and B show different target specificity (*FvZBD1* or *FvABC3*) and synergistic action. These findings will help inform the optimization of maximally efficacious *S. zeae* strains for eliminating *F. verticillioides* colonization and fumonisin contamination in maize cropping systems. This novel study contributes significantly to our knowledge of competitive microorganism relationships and the role of secondary metabolites in antagonistic fungal-fungal interactions.

## 1 Introduction

*Fusarium verticillioides* is a mycotoxigenic fungus ubiquitously associated with maize worldwide (Presello et al., 2008; Baldwin et al., 2014; Blacutt et al., 2018). As a prolific, soilborne, primary pathogen of maize, *F. verticillioides* is capable of causing disease at all plant growth stages, including seedling blight, and root, stalk, ear, and kernel rots (Brown et al., 2008). *F. verticillioides* is of primary concern because it produces secondary metabolites—the fumonisin mycotoxins—that accumulate in the grain and are toxic to animals (Duncan and Howard, 2010). Exacerbating its importance, maize is a global staple for human populations and a critical component of livestock feed. *F. verticillioides* frequently exists as an asymptomatic, yet mycotoxigenically active endophyte of maize, underscoring the persistence of fumonisin contamination (Duncan and Howard, 2010). Since this fungus is ubiquitous in maize cultivation, innovative solutions to eliminate fumonisin contamination are vital for agricultural economics, global food and feed safety, and the health and welfare of livestock and humans.

Fumonisins are polyketide secondary metabolites produced predominantly by select members of the *Fusarium fujikuroi* species complex, including *F. verticillioides* (Rheeder et al., 2002). In maize, fumonisins occur mainly in series B, where fumonisin B_1_ (FB_1_) is produced the most abundantly and is the most toxic, followed by decreasing levels and toxicity of fumonisin B_2_ (FB_2_), B_3_ (FB_3_), and B_4_ (FB_4_) (Proctor, 2000).

Fumonisins are structurally analogous to sphinganine, the backbone and precursor molecule of sphingolipids, and most notably, ceramide (Proctor, 2000). Sphingolipids are essential structural components of eukaryotic cell membranes and signaling molecules (Young et al., 2012). Fumonisins impede sphingolipid metabolism by inhibiting ceramide synthase (a key enzyme in sphingolipid biosynthesis) via competition with sphinganine (Merrill et al., 1993). Disruption of this pathway leads to the accumulation of free sphinganine and the reduction in ceramide-based, complex sphingolipids (Merrill et al., 1993; Blacutt et al., 2018).

Through sphingolipid metabolism disruption, fumonisins induce several animal diseases, including equine leukoencephalomalacia, porcine pulmonary edema, and hepato- and nephrotoxicity in rats and mice (Blacutt et al., 2018). In human populations, epidemiological evidence associated high consumption of fumonisin-contaminated maize with esophageal cancer (Marasas et al., 1981), neural tube birth defects (through maternal consumption) (Gelineau-van Waes et al., 2009), and growth impairment in children (Shirima et al., 2015). In *F. verticillioides*-maize infections, FB_1_ is phytotoxic and plays a significant role in seedling disease development and disease severity in sensitive maize lines (Glenn et al., 2008).

*Sarocladium zeae* is an endophytic fungus that colonizes and frequently coinhabits maize kernels with *F. verticillioides*. *S. zeae* produces pyrrocidines A and B, which are lactam-containing, secondary metabolites previously described for their antibacterial (e.g., *Staphylococcus* spp. and *Enterococcus* spp.) (He et al., 2002) and antifungal (e.g., *Aspergillus flavus* and *F. verticillioides*) properties (Wicklow et al., 2005; Wicklow and Poling, 2009). Pyrrocidine A and B differ by the presence of a double bond between carbons 17 and 18 in their lactam ring (He et al., 2002). This subtle, structural difference confers pyrrocidine A with higher toxicity to microbes and mammalian cell lines than pyrrocidine B (Wicklow et al., 2005; Haschek et al., 2008; Wicklow and Poling, 2009; Uesugi et al., 2016). Pyrrocidines can be detected in *S. zeae*-colonized kernels (Wicklow and Poling, 2009), yet their natural occurrence and quantity remain unreported. *S. zeae* does not induce disease symptoms in the plant, supporting its role as a common, natural, protective endophyte in maize, and strengthening its potential as a biological control agent (Gao et al., 2020).

Gao et al. (2020) investigated *F. verticillioides*’ physiological response to pyrrocidines. They showed that pyrrocidine B (the only pyrrocidine treatment assessed) inhibited the growth of *F. verticillioides*. Importantly, at subinhibitory levels for growth, pyrrocidine B reduced FB_1_ levels by 99.5% and eliminated detectable FB_2_ and FB_3_ production. In response to pyrrocidine B challenge, *FvZBD1* (FVEG_00314), encoding a putative zinc-binding dehydrogenase, was the most highly induced gene at over 4,000-fold. *FvZBD1* is located directly adjacent to the fumonisin biosynthetic gene cluster in *F. verticillioides* (FVEG_00315 to FVEG_00329) (Brown et al., 2012). Deletion of *FvZBD1* resulted in dramatic increases in fumonisin production (FB_1_ >30-fold, FB_2_ >40-fold, FB_3_ >8-fold) and enhanced virulence in fumonisin-sensitive maize seedlings. Trans-complementation of *FvZBD1* into Δ*FvZBD1* rescued all mutant phenotypes. Thus, *FvZBD1* acts as a repressor of fumonisin biosynthesis and can be considered part of the fumonisin biosynthetic regulatory machinery. Interestingly, Δ*FvZBD1* also exhibited slightly reduced growth in response to 10 μg/mL pyrrocidine B challenge. An additional gene of interest, *FvABC3* (FVEG_11089), encoding an ABC transporter, was upregulated over 400-fold in response to pyrrocidine B. Δ*FvABC3* had elevated sensitivity to 10 μg/mL pyrrocidine B, presenting as strong growth inhibition. This suggests that *FvABC3* is required for pyrrocidine tolerance.

Creating innovative solutions to mitigate fumonisin contamination in maize is vital for food and feed safety and the health of livestock and humans. As previously described, *S. zeae* holds strong potential as a biocontrol agent to dually reduce *F. verticillioides* growth and fumonisin contamination in the field. *S. zeae* strains exhibit variable pyrrocidine production, per chemotype, varying in pyrrocidine compound bias (e.g., making just pyrrocidine A, B, or both pyrrocidine A and B) or amount produced (Wicklow et al., 2008). The molecular mechanisms driving pyrrocidine-induced growth inhibition and fumonisin elimination are unknown. Further, comprehensive pyrrocidine dose-response studies—including pyrrocidine A, B, and uniquely examining the combined effect of pyrrocidine A + B—have not been conducted in *F. verticillioides* (or any microorganism to date). To investigate the biocontrol potential of *S. zeae* and the role of pyrrocidine, we must first understand how pyrrocidine inhibits growth and fumonisin production. The first step in this understanding is to explore the dose-dependent response of *F. verticillioides* strains to pyrrocidines. Beyond a descriptive study, it is critical to identify the lowest possible dosage of pyrrocidine that can eliminate *F. verticillioides* growth. This study is the first comprehensive report examining the dose-dependent response of *F. verticillioides* to pyrrocidine A, B, and A + B. Here, the combined effect of pyrrocidine A + B was explored and determined for the first time.

## 2 Materials and methods

### 2.1 Chemicals and media

Purified pyrrocidine A and B compounds were obtained from Bioaustralis Fine Chemicals (Smithfield, New South Wales, Australia). Dimethyl sulfoxide (DMSO)—used as the chemical carrier/solvent for pyrrocidine—was purchased from Sigma-Aldrich, Inc. (Saint Louis, MO, United States). Potato dextrose broth (PDB; Neogen, Lansing, MI, United States) and modified *Fusarium* minimal media (mFMM) were used as growth media in this study. mFMM was prepared as described in the *Fusarium* Laboratory Manual (Leslie and Summerell, 2006), with slight modifications (Bueschl et al., 2014). In brief, mFMM was composed of: 1 g/L KH_2_PO_4_; 0.5 g/L MgSO_4_*·*7H_2_O; 0.5 g/L KCl; 2 g/L NaNO_3_; 0.2 mL/L trace element solution (5 g citric acid; 5 g ZnSO_4_*·*6H_2_O; 1 g Fe(NH_4_)_2_(SO_4_)_2_*·*6H_2_O; 250 mg CuSO_4_*·*5H_2_O; 50 mg MnSO_4_; 50 mg H_3_BO_3_; 50 mg Na_2_MoO_4_*·*2H_2_O; nominal 100 mL with distilled water); and modified with 30 g/L glucose.

### 2.2 Fungal strains and growth conditions

Strains of *F. verticillioides* used in this study are listed in Table 1. To note, Gao et al. (2020) confirmed that the Δ*FvZBD1* and Δ*FvABC3* complemented strains—Δ*FvZBD1*-1::C2 (FVEG_00314-4-1::3-2) and Δ*FvABC3*-2::C1 (FVEG_11089-15-1::3-2)—restored wild type-like phenotypes in fumonisin production, pyrrocidine sensitivity, and fungal virulence. Thus, for simplicity, we have only assessed the deletion strains here. Fungal strains were routinely cultured from frozen stocks in PDB, in a dark incubator, at 27°C, 250 rpm, for 4 days. Cells were harvested from liquid cultures, purified, quantified using a LUNA^™^ Automated Cell Counter (Logos Biosystems, Aligned Genetics, Inc., Annandale, VA, United States), and used immediately as experimental inoculum. Pyrrocidine dose-response assays were conducted in mFMM to remain consistent with future metabolomic experiments designed to further investigate the molecular mechanisms of pyrrocidine-based fumonisin inhibition.

**Table 1:** Strains of *F. verticillioides* used in this study.

Strain	Genotype	Description	Source
FRC^a^ M-3125	Wild type	*F. verticillioides* wild-type strain (reference strain 7600)	Ma et al. (2010)
RRC^b^ 2954 (FVEG_00314-4-1)	Δ*FvZBD1*-1	*F. verticillioides* FVEG_00314 deletion mutant, in FRC M-3125 background	Gao et al. (2020)
RRC 2951 (FVEG_11089-15-1)	Δ*FvABC3*-2	*F. verticillioides* FVEG_11089 deletion mutant, in FRC M-3125 background	Gao et al. (2020)

a FRC, *Fusarium* Research Center, Pennsylvania State University.

b RRC, Russell Research Center, now known as the U.S. National Poultry Research Center, USDA, ARS, Athens, GA.

### 2.3 Pyrrocidine dose-response assay

Growth curve analyses were conducted to determine the dose-dependent response of *F. verticillioides* strains to pyrrocidines, and to evaluate the treatment effect of pyrrocidine A + B in combination. Analyses were completed using a Bioscreen C (Growth Curves USA, Piscataway, NJ, United States). The growth of *F. verticillioides* wild-type (WT), Δ*FvZBD1*, and Δ*FvABC3* strains was assessed under the following treatment conditions: no-treatment control; 0.5% DMSO control; 0.5% DMSO containing either pyrrocidine A, B, or combination A + B at the following final, total concentrations, (1) 0.5, (2) 1.0, (3) 2.5, (4) 5.0, (5) 10, and (6) 20 μg/mL (Supplementary Table S1). For the pyrrocidine A + B combination treatments, pyrrocidine A and B were each combined at half the reported final concentration (e.g., 20 μg/mL pyrrocidine A + B = 10 μg/mL pyrrocidine A + 10 μg/mL pyrrocidine B). All treatments contained 0.5% DMSO. Experiments were completed a minimum of three times, with similar results obtained from each experimental replicate. Supplementary Figure S1 depicts the experimental workflow. In brief, a series of master mixes were prepared. First, a master mix of *F. verticillioides* conidia and growth media was prepared per strain, at 2 × 10^4^ conidia/mL mFMM. Second, stocks were prepared per pyrrocidine compound and treatment dose. Stocks were combined with the cells and media to generate a master mix of the appropriate treatment conditions. Each treatment condition master mix was then distributed into Bioscreen honeycomb microtiter plates (Growth Curves Ab Ltd., Helsinki, Finland), with 10 technical replicates per treatment condition. Microtiter plates were incubated for 5 days (120 h) in the dark, at 28°C, with continuous shaking. In these conditions, *F. verticillioides* undergoes microconidiation (Glenn et al., 2004) and grows as a near-pure culture of conidia (i.e., yeast-like growth) (Gao et al., 2020). Thus, optical density (OD) readings are similar to those of yeast cultures (Gao et al., 2020), and are an accurate measure of growth. Fungal growth measurements (as OD A600nm) were recorded every 30 min.

### 2.4 Defining the sensitivity of *Fusarium verticillioides* wild-type to pyrrocidine A + B between 10 and 20 μg/mL

Upon completion of the pyrrocidine dose-response assay, as described in Supplementary Table S1 and Supplementary Figure S1, there was a large growth discrepancy (e.g., growth versus no growth) noted between the 10 and 20 μg/mL pyrrocidine A + B combination treatment doses. Thus, a supplementary pyrrocidine dose-response assay was conducted on *F. verticillioides* WT to refine and document its sensitivity, using the following treatment conditions: no-treatment control; 0.5% DMSO control; 0.5% DMSO containing pyrrocidine A + B combined at the following final concentrations, (1) 10, (2) 12, (3) 14, (4) 16, (5) 18, and (6) 20 μg/mL (Supplementary Table S2). This experiment was conducted according to the above *pyrrocidine dose-response assay* in materials and methods section 2.3.

### 2.5 Fumonisin quantification

After the pyrrocidine dose-response assay concluded (at 120 h), Δ*FvZBD1* samples were analyzed via UHPLC-MS (ultra-high-performance liquid chromatography-mass spectrometry) for fumonisin (FB_1_, FB_2_, FB_3_) detection and quantification. To extract fumonisins from the whole liquid culture, 4 replicates were tested per treatment [each consisting of 2 wells combined out of 10 (excluding the edge wells)], and an equal volume of a 100% acetonitrile (Honeywell, ≥ 99.9%) + 5% formic acid (Mallinckrodt, 88%) solution was added to each sample. The extracts were thoroughly vortexed and incubated overnight at 4°C. If necessary, samples in the extraction solution were stably stored at 4°C for future processing (acetonitrile kills fungal cells and denatures enzymes, and fumonisins remain stable). Extracts were spin filtered (Corning Costar Spin-X Centrifuge Tube Filters, Neta Scientific, Marlton, NJ, United States) for 2 min at maximum speed (20,817 rcf) to remove the fungal tissue and culture debris, diluted 1:1 with distilled water, and analyzed via UHPLC-MS (Dionex UltiMate 3000; Thermo LTQ-XL) for fumonisin content as previously described (Gao et al., 2020).

### 2.6 Statistical analysis of pyrrocidine dose-response assay data

#### 2.6.1 Model fitting

We fit a Bayesian generalized additive mixed model (GAMM) (Wood, 2017) to the OD (growth) data. The response variable was OD on a logarithmic scale. The model included a spline term to flexibly fit the logarithmic OD trends over time, with fixed effects for compound, concentration (treated as a discrete variable), strain, and all two-way and three-way interactions, and random intercepts for plate and well nested within a plate. The spline term was a factor-smooth interaction grouped by the interaction of compound, concentration, and strain, with basis dimension *k* = 5 and first-order penalty. For computational reasons, we fit the model to only 1 in every 10 time points (every 5 h, where the original data were measured every 30 min). We assumed normal priors with mean 0 and standard deviation 1 for all fixed effect coefficients; this is a weakly informative prior that constrains effect sizes to a plausible range and speeds model convergence. The joint posterior distribution of all parameters was sampled using the Hamiltonian Monte Carlo algorithm; we ran four Markov chains, each for 3,000 discarded warmup iterations and 2000 post-warmup sampling iterations, for a total of 5,000 posterior samples. We assessed model convergence by examining trace plots and confirming that the potential scale reduction factor $\hat{R}\leq 1.01$ for all parameters, and we assessed model fit by examining a posterior predictive check plot.

#### 2.6.2 Model predictions

We calculated the expected value of the posterior for all tested combinations of compound, concentration, and strain at 5-h intervals from 0 to 120 h. We also calculated posterior estimates for the maximum growth rate (by taking the maximum slope of log OD versus time from the fitted spline curves), final growth (by estimating the posterior expected value of log OD at the final time point, 120 h), and the area under the log OD growth curve (estimated by trapezoidal integration of the expected values of the posterior at each time point). Means for maximum growth rate, final growth, and area under the growth curve were compared between concentrations within each combination of compound and strain, by taking the posterior distribution of the differences between concentrations and calculating the Bayesian *p*_MAP_ (maximum *a posteriori p*-value) (Makowski et al., 2019) compared to a null value of 0 difference. We considered *p*_MAP_ values <0.05, which correspond to a 20-fold or greater ratio of posterior probability between the observed difference and the null value, to be statistically significant. Means were also compared between strains within each combination of compound and concentration, and between all combinations of compound and concentration within each strain. For all comparisons that involved comparing 0 concentration to other concentrations of a compound, we used the posterior prediction from the DMSO control treatment to represent 0 concentration. We also compared values for the no-treatment control and DMSO control treatment within each strain. In all cases, we present the median value of the distribution of the expected value of the posterior predictive distribution, along with equal-tailed quantile credible intervals to show uncertainty.

#### 2.6.3 Software

We used R software v4.3.1 (R Core Team, 2023) and Stan software v2.33.1 (Stan Development Team, 2023) to analyze the data, including the R packages brms v2.20.5 (Bürkner, 2018), cmdstanr v0.7.0 (Gabry and Češnovar, 2023), mgcv v1.8-42 (Wood, 2004), tidybayes v3.0.4 (Kay, 2022), and bayestestR v0.13.1 (Makowski et al., 2019).

### 2.7 Statistical analysis of Δ*FvZBD1* fumonisin data

The data from the pyrrocidine dose-response assay conducted on Δ*FvZBD1* from a single experimental replicate were analyzed following a completely randomized design. We fit a separate linear model for each of the three types of fumonisins (FB_1_, FB_2_, and FB_3_). In each case, the response variable was log-transformed, being a concentration that is strictly positive and right-skewed. The treatment effect had 20 discrete levels: no-treatment control, DMSO control, and six concentration levels (0.5, 1, 2.5, 5, 10, and 20 μg/mL) for each of the three pyrrocidine treatments (A, B, and A + B). Separate post-hoc tests were done within each pyrrocidine treatment to compare mean fumonisin biosynthesis, adjusting the *p*-values using the Tukey adjustment. The mean from the DMSO control was used within each test to represent a concentration of 0. In addition, separate post-hoc tests were done to compare the means between the no-treatment control and the DMSO control.

## 3 Results

### 3.1 0.5% DMSO does not significantly affect *Fusarium verticillioides* growth

**Figure 1:**
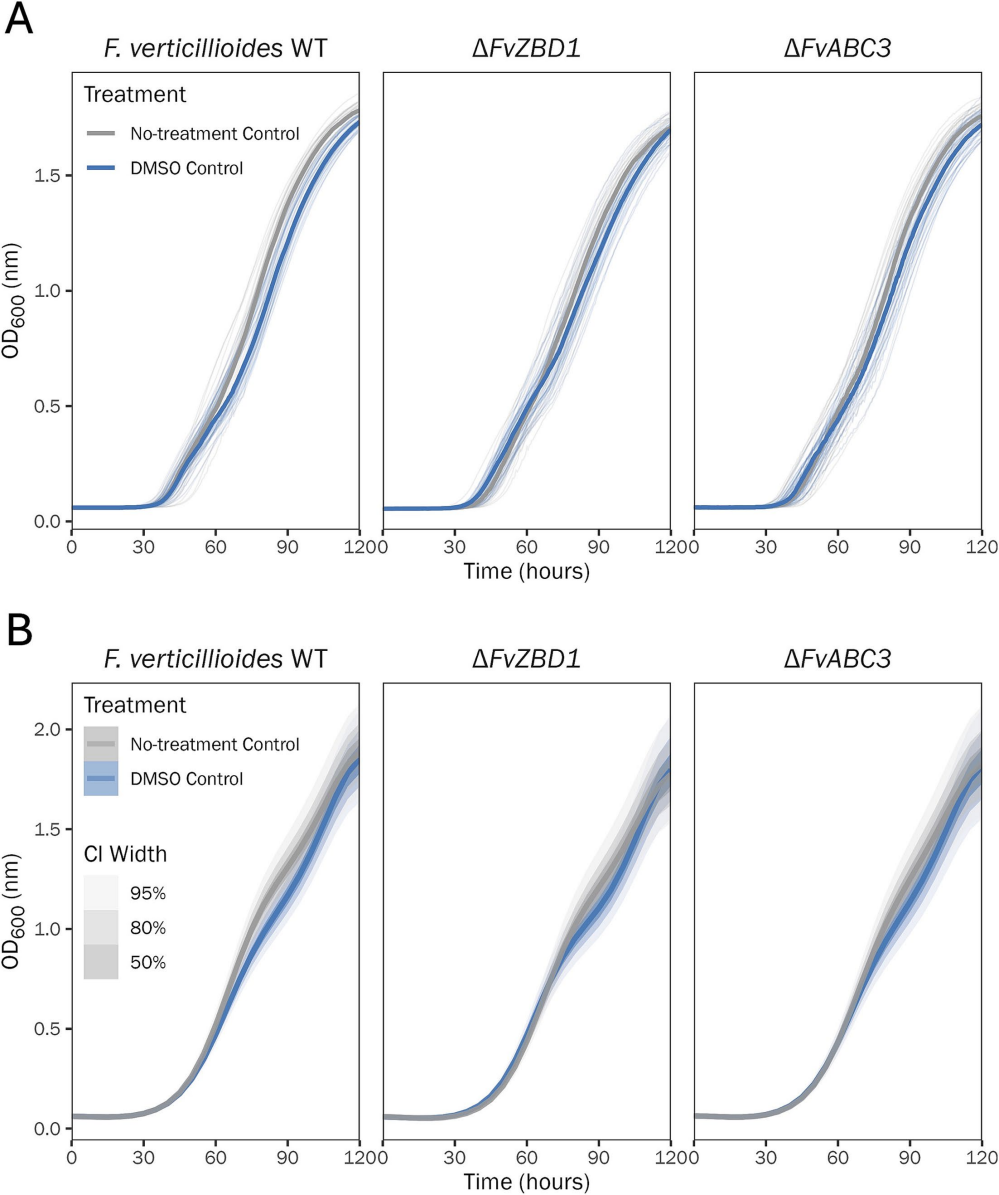
0.5% DMSO does not significantly affect *F. verticillioides* growth curves. Growth curve analysis of the *F. verticillioides* WT, Δ*FvZBD1*, and Δ*FvABC3* no-treatment control and 0.5% DMSO control, monitored for 120 h, at 28°C, with continuous shaking in the dark in modified *Fusarium* minimal media. Ten replicates were tested per treatment, and optical density (OD600) measurements were recorded every 30 min. **(A)** Plot of the no-treatment control and DMSO control raw data. Individual wells (replications) are plotted as thin semi-transparent lines, and medians are plotted as thick lines. **(B)** Plot of the no-treatment control and DMSO control fitted model values. Medians of the posterior distributions are plotted as thick lines, with credible intervals shown as progressively lighter-shaded areas. CI, credible interval.

DMSO was the solvent used for all pyrrocidine compounds. As such, it was first essential to ensure that the DMSO dosage did not significantly affect *F. verticillioides* growth. To assess this potential interaction, a no-treatment control and DMSO control were used for all experiments. All pyrrocidine treatments, including the DMSO control, contained 0.5% DMSO. Growth curve analyses identified that there was no meaningful statistical or biologically relevant difference in total growth between the no-treatment control and DMSO control (Figure 1 and Supplementary Table S3).

### 3.2 Evidence for a potent dose-dependent synergy between pyrrocidines A and B

**Figure 2:**
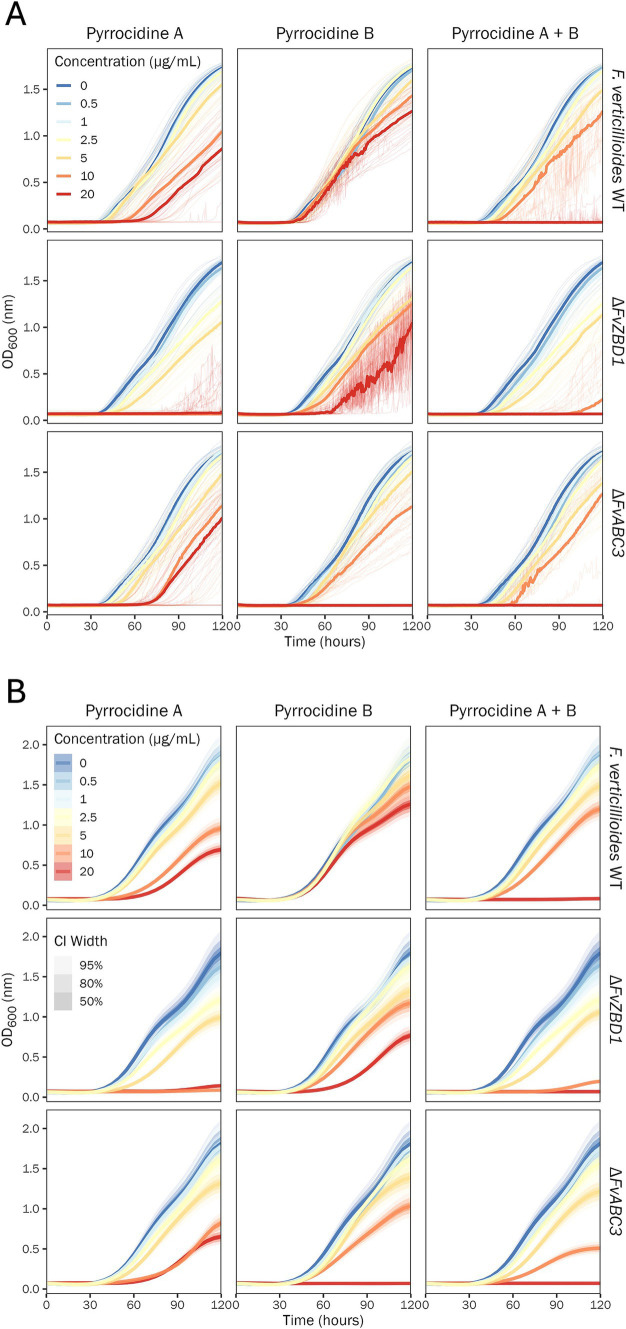
Pyrrocidine A, B, and combination A + B inhibit *F. verticillioides* growth in a dose-, compound-, and strain-dependent manner. Growth curve analysis of *F. verticillioides* WT, Δ*FvZBD1*, and Δ*FvABC3* strains challenged with the following treatment conditions: 0.5% DMSO control; 0.5% DMSO containing either pyrrocidine A, B, or combination A + B at the following final concentrations, (1) 0.5, (2) 1.0, (3) 2.5, (4) 5.0, (5) 10, and (6) 20 μg/mL, monitored for 120 h, at 28°C, with continuous shaking in the dark in modified *Fusarium* minimal media. Ten replicates were tested per treatment, and optical density (OD600) measurements were recorded every 30 min. **(A)** Plot of the raw data. Individual wells (replications) are plotted as thin semi-transparent lines, and medians are plotted as thick lines. **(B)** Plot of the fitted model values. Medians of the posterior distributions are plotted as thick lines, with credible intervals shown as progressively lighter-shaded areas. CI, credible interval.

To investigate the dose-dependent response of *F. verticillioides* WT, Δ*FvZBD1*, and Δ*FvABC3* to pyrrocidine A, B, and A + B in combination, pyrrocidine-dose response assays were conducted to assess *F. verticillioides* growth over time. *F. verticillioides* strains were challenged with the following treatment conditions: no-treatment control; 0.5% DMSO control; 0.5% DMSO containing either pyrrocidine A, B, or combination A + B at the following final concentrations, (1) 0.5, (2) 1.0, (3) 2.5, (4) 5.0, (5) 10, and (6) 20 μg/mL. Previous findings showed that pyrrocidines inhibit *F. verticillioides* growth (Wicklow et al., 2005; Wicklow and Poling, 2009; Gao et al., 2020). Here, the inhibition of wild type growth increased proportionately as pyrrocidine A and B concentrations increased, up to the maximum concentration of 20 μg/mL (Figure 2). At the maximum dosage of both pyrrocidine A and B, wild-type cells were viable and growing, yet at an inhibited growth rate and to a lower final OD value. In accordance with previous literature, pyrrocidine A exhibited a dose-dependent higher toxicity than pyrrocidine B—marked by a significantly lower fungal growth rate (Supplementary Table S4), total growth (area under the OD growth curve; Supplementary Table S5), and final OD value (Supplementary Table S6), at approximately 5 μg/mL pyrrocidine A or higher.

Remarkably, growth curve analyses provided newfound evidence for a potent dose-dependent synergy between pyrrocidines A and B, meaning that the combined compounds were more effective at inhibiting *F. verticillioides* WT growth than the individual compounds (Figure 2). When combined, pyrrocidine A + B inhibited wild-type cells proportionately more until a threshold above 10 μg/mL, where growth was eliminated at 20 μg/mL (20 μg/mL pyrrocidine A + B = 10 μg/mL pyrrocidine A + 10 μg/mL pyrrocidine B; Supplementary Table S1).

**Figure 3:**
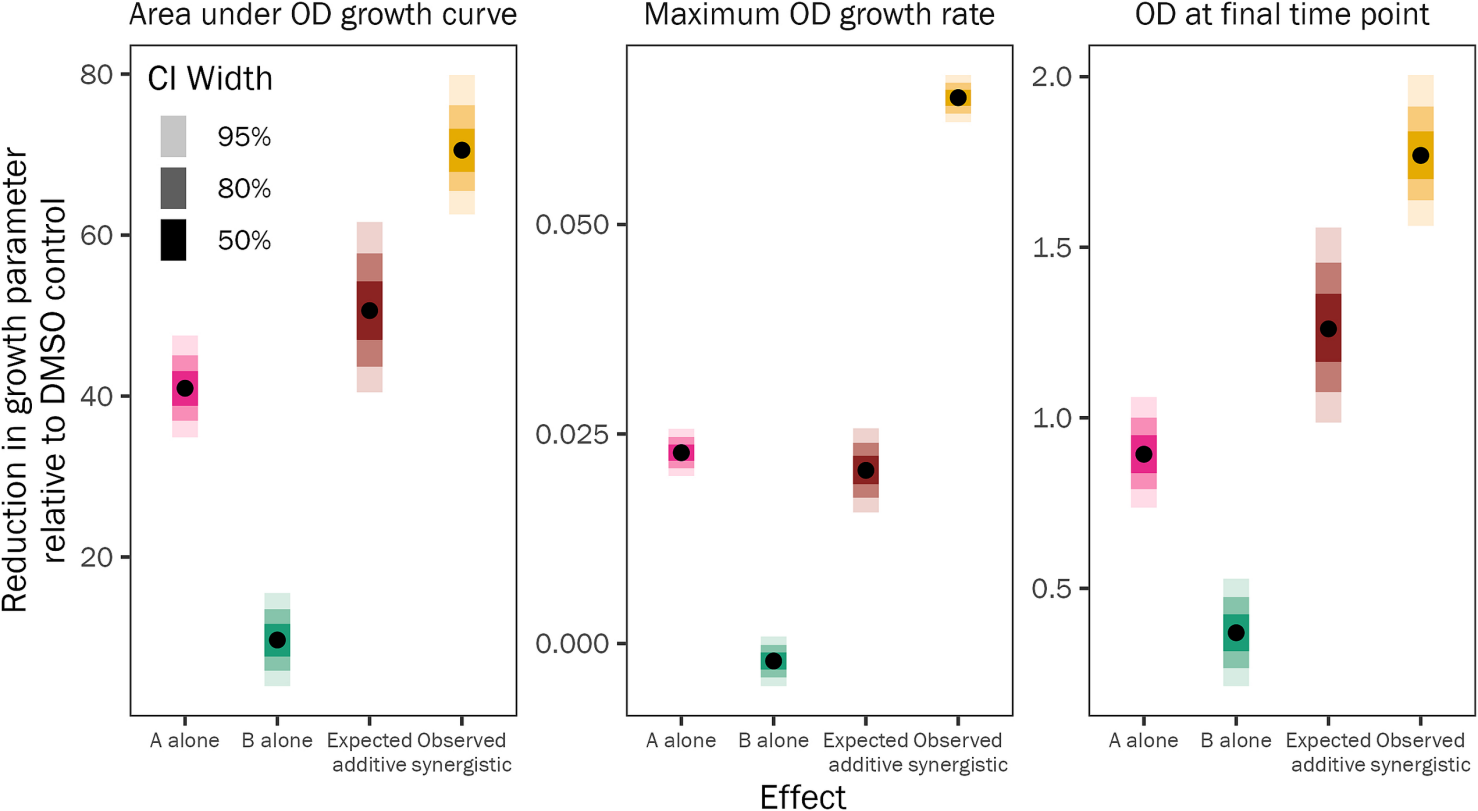
Pyrrocidines A and B act synergistically to inhibit *F. verticillioides* WT growth. Plot of the fitted model values illustrating the mean reduction in *F. verticillioides* WT growth from 10 μg/mL pyrrocidine A alone (pink) or 10 μg/mL pyrrocidine B alone (green). The expected additive effect (brown) describes the expected, calculated inhibition of 20 μg/mL pyrrocidine A + B if pyrrocidine A and B acted additively to reduce growth. This expected additive value is the sum of two differences: the difference between the DMSO control mean and the 10 μg/mL pyrrocidine A mean; plus, the difference between the DMSO control mean and the 10 μg/mL pyrrocidine B mean. The observed synergistic value (yellow) represents the observed reduction in growth from 20 μg/mL pyrrocidine A + B, which is significantly higher than the expected additive value for each growth parameter [total growth (area under the optical density (OD) growth curve), maximum OD growth rate, and final OD (OD at the final time point)]. This means that pyrrocidines A and B act synergistically—not additively—to inhibit *F. verticillioides* WT growth. Medians of the posterior estimates of the growth parameters are shown as points and credible intervals as progressively lighter-shaded bars. CI, credible interval.

We examined the pyrrocidine A + B combined effect to decipher if the inhibition phenotype was synergistic or additive. To assess this, we compared the observed growth inhibition of 20 μg/mL pyrrocidine A + B (growth in the DMSO control minus growth in 20 μg/mL pyrrocidine A + B) to the expected combined growth inhibition of 10 μg/mL pyrrocidine A and 10 μg/mL pyrrocidine B [(growth in DMSO control minus growth in 10 μg/mL pyrrocidine A) plus (growth in DMSO control minus growth in 10 μg/mL pyrrocidine B)] (Figure 3 and Supplementary Table S7). The observed inhibition from 20 μg/mL pyrrocidine A + B was significantly greater than the expected inhibition from 10 μg/mL pyrrocidine A plus that of 10 μg/mL pyrrocidine B, meaning that this interaction was synergistic, not additive. Pyrrocidine A + B synergistically inhibited *F. verticillioides* WT maximum growth rate, total growth, and final OD approximately 3.15, 1.39, and 1.40 times greater, respectively, than the calculated additive effect (Figure 3 and Supplementary Table S7). Thus, the growth inhibition from the pyrrocidine A + B combination treatment represented true synergistic activity.

Of importance, the pyrrocidine A + B combination dose delivered the same final in-culture concentration of total pyrrocidine as the single compound treatments (Supplementary Table S1). This means that the synergistic growth inhibition phenotype—comparing 20 μg/mL pyrrocidine A + B and 10 μg/mL pyrrocidine A + 10 μg/mL pyrrocidine B—is compound-based, not concentration-based. While 20 μg/mL pyrrocidine A + B eliminated growth, containing 10 μg/mL pyrrocidine A plus 10 μg/mL pyrrocidine B, neither 20 μg/mL pyrrocidine A nor 20 μg/mL pyrrocidine B eliminated growth, substantiating the potency of pyrrocidine A + B’s synergistic activity in inhibiting *F. verticillioides* growth.

**Figure 4:**
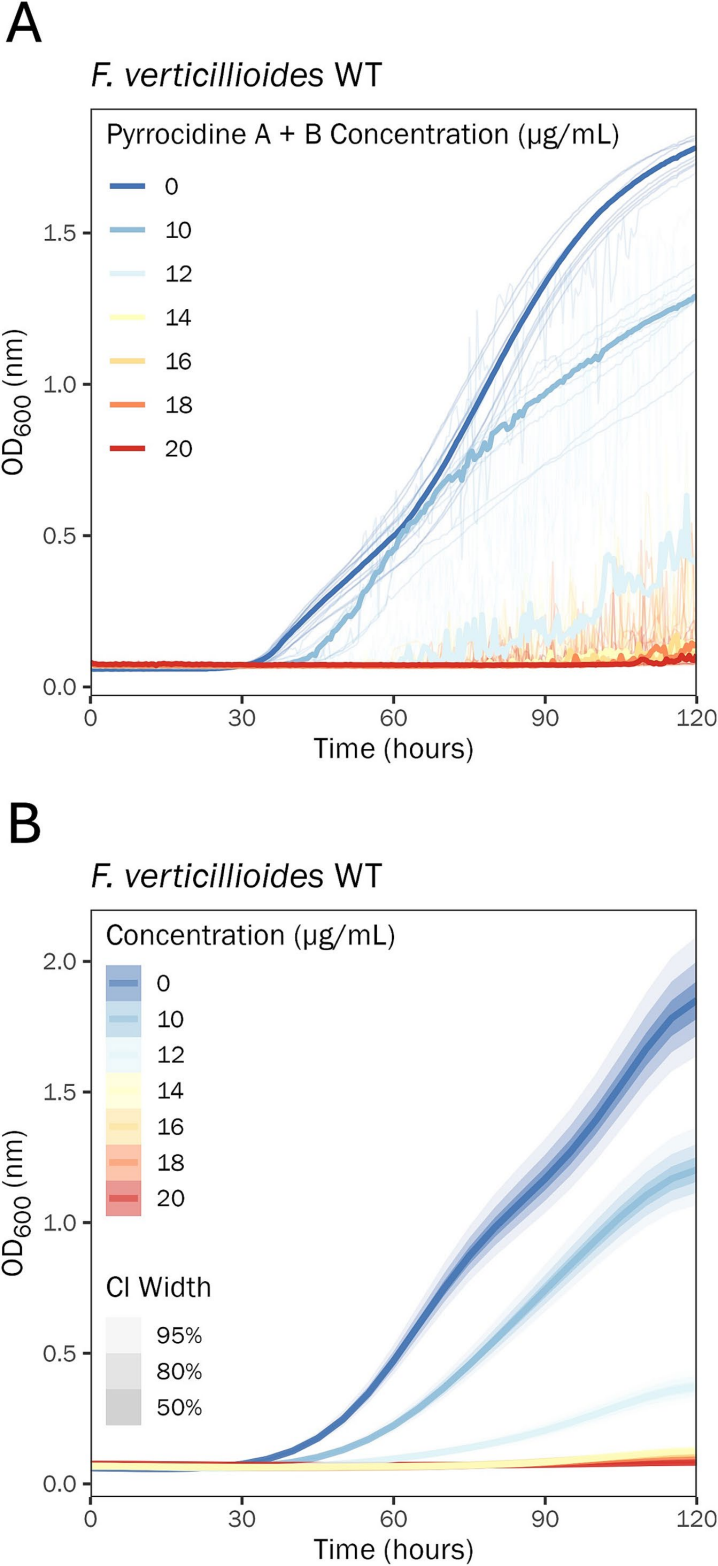
14 μg/mL pyrrocidine A + B in combination nearly eliminates *F. verticillioides* WT growth. Growth curve analysis of *F. verticillioides* WT challenged with the following treatment conditions: 0.5% DMSO control; 0.5% DMSO containing pyrrocidine A + B combined at the following final concentrations, (1) 10, (2) 12, (3) 14, (4) 16, (5) 18, and (6) 20 μg/mL, monitored for 120 h, at 28°C, with continuous shaking in the dark in modified *Fusarium* minimal media. Ten replicates were tested per treatment, and optical density (OD600) measurements were recorded every 30 min. **(A)** Plot of the raw data. Individual wells (replications) are plotted as thin semi-transparent lines, and medians are plotted as thick lines. **(B)** Plot of the fitted model values. Medians of the posterior distributions are plotted as thick lines, with credible intervals shown as progressively lighter-shaded areas. CI, credible interval.

There was a large growth discrepancy (e.g., growth versus no growth) noted between the 10 and 20 μg/mL pyrrocidine A + B combination treatment doses. To resolve this gap, *F. verticillioides* WT was challenged with 0.5% DMSO containing pyrrocidine A + B at the following final concentrations: (1) 10, (2) 12, (3) 14, (4) 16, (5) 18, and (6) 20 μg/mL. Pyrrocidine A + B inhibited wild type growth progressively more until a threshold concentration between 12 and 14 μg/mL, above which it inhibited growth almost entirely (Figure 4).

To note, at the doses tested here, pyrrocidine acted in a fungistatic manner, consistent with the findings of Wicklow and Poling (2009). After the 120-h pyrrocidine dose-response assay, all cells, including those in inhibitory treatments that exhibited no growth (e.g., 20 μg/mL pyrrocidine A + B), were plated on mFMM solid media and grew without defect (data not shown).

Growth inhibition patterns were similar whether we compared the maximum growth rate, total growth (area under the OD growth curve), or OD at the final time point (120 h) (Supplementary Figures S2–S4).

### 3.3 *FvZBD1* confers partial tolerance to pyrrocidines

Growth curve analyses revealed that the *F. verticillioides* Δ*FvZBD1* mutant exhibited extreme sensitivity to pyrrocidine A and elevated sensitivity to pyrrocidine B, as compared to *F. verticillioides* WT (Figure 2 and Supplementary Table S8). Inhibition of Δ*FvZBD1* growth increased proportionately as pyrrocidine A dose increased, until 5 μg/mL. Between 5 and 10 μg/mL pyrrocidine A, a threshold effect occurred, where 10 μg/mL and above eliminated growth. For pyrrocidine B, there was no threshold effect for Δ*FvZBD1*, and growth inhibition qualitatively resembled that of pyrrocidine A in wild-type cells. However, under pyrrocidine B challenge, Δ*FvZBD1* presented a stress-induced irregular growth phenotype, generating mycelial balls in shaking liquid cultures, rather than growing as a uniform conidial suspension as in wild type (Supplementary Figure S5). The OD measurement of these mobile mycelial balls generated jagged growth curves and increased (high) variance, as seen in the raw data (Figure 2A; Supplementary Figure S6). Pyrrocidine A + B in combination had a similar effect to pyrrocidine A in Δ*FvZBD1*. Growth inhibition increased continuously until a threshold between 5 and 10 μg/mL. Pyrrocidine A + B at 10 μg/mL inhibited growth nearly completely, and 20 μg/mL eliminated growth. Growth inhibition and phenotypes could be clearly visualized in the microtiter culture plate at 120 h (Supplementary Figure S5).

### 3.4 Pyrrocidine functions through *FvZBD1* to effectively eliminate fumonisin biosynthesis

**Figure 5:**
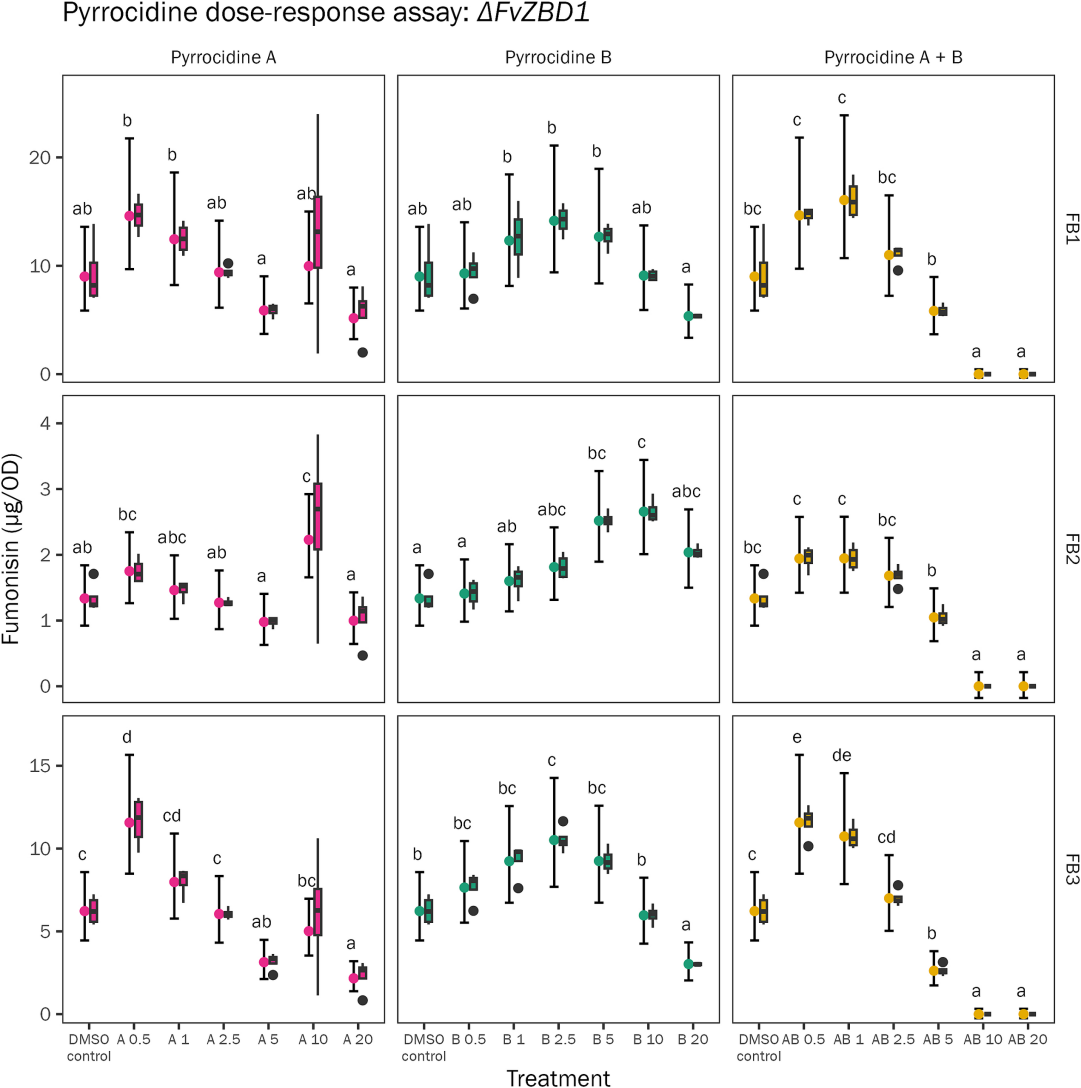
*F. verticillioides* Δ*FvZBD1* mutant fumonisin biosynthesis was generally not inhibited by pyrrocidine challenge. The *F. verticillioides* Δ*FvZBD1* mutant was challenged with the following treatment conditions: 0.5% DMSO control; 0.5% DMSO containing either pyrrocidine A, B, or combination A + B at the following final concentrations, (1) 0.5, (2) 1.0, (3) 2.5, (4) 5.0, (5) 10, and (6) 20 μg/mL, and monitored for 120 h, at 28°C, with continuous shaking in the dark in modified *Fusarium* minimal media. Plots represent Δ*FvZBD1* fumonisin production in all treatment conditions, normalized to growth [optical density (OD600)]. Data was collected at 120 h, following the conclusion of the pyrrocidine dose-response assay. Data from a single experimental replicate is presented. The graph shows the distribution of the raw data (as box and whisker plots) and the means with 95% confidence intervals for fumonisin type (FB_1_, FB_2_, or FB_3_) and concentration per treatment. The multiple comparison letters, for each combination of fumonisin and pyrrocidine, are valid within each panel.

As previously described by Gao et al. (2020), pyrrocidine B was very effective at reducing FB_1_, FB_2_, and FB_3_ production in *F. verticillioides* WT—where fumonisin inhibition increased linearly as pyrrocidine dose increased, and 5 μg/mL pyrrocidine B reduced FB_1_ levels by 99.5% and eliminated detectable FB_2_ and FB_3_ production. Here, the *F. verticillioides* Δ*FvZBD1* mutant’s fumonisin production was measured at the conclusion of the pyrrocidine dose-response assay (120 h) to assess how pyrrocidines impact Δ*FvZBD1* fumonisin biosynthesis—a previously unassessed and unknown interaction. Δ*FvZBD1* fumonisin production was not linearly related to pyrrocidine treatment, as observed in wild type (Figure 5). Rather, we observed a high variation in fumonisin production following each pyrrocidine challenge. FB_1_ production was not significantly different between the DMSO control and pyrrocidine treatments. FB_2_ had little to no differences between the control and pyrrocidine treatments as well, yet interestingly showed elevated production under select treatments (e.g., 10 μg/mL pyrrocidine B). FB_3_ biosynthesis was variable, where some treatments produced higher or lower FB_3_ levels. To note, treatments that approached complete growth inhibition in all replicates (e.g., 10 and 20 μg/mL pyrrocidine A + B) produced little-to-no detectable fumonisins, accordingly. Importantly, fumonisin biosynthesis continued to be induced in Δ*FvZBD1* under all pyrrocidine challenges, in a pyrrocidine dose-independent manner. Thus, fumonisin biosynthesis in Δ*FvZBD1* was generally not inhibited in response to pyrrocidine challenge (Figure 5; Supplementary Figure S7). This data provides evidence that pyrrocidine functions through *FvZBD1* to repress fumonisin production in *F. verticillioides* WT.

### 3.5 *FvABC3* has functional specificity to pyrrocidine B

In the pyrrocidine dose-response assay, the *F. verticillioides* Δ*FvABC3* mutant exhibited wild type-like growth under pyrrocidine A challenge, yet had elevated sensitivity to pyrrocidine B (Figure 2 and Supplementary Table S11). Inhibition of Δ*FvABC3* growth increased proportionately as pyrrocidine A concentration increased, up to the maximum concentration of 20 μg/mL. As pyrrocidine B dose increased, growth was inhibited continuously until a threshold was reached between 10 and 20 μg/mL—where growth was completely inhibited at 20 μg/mL. Pyrrocidine A + B in combination had a similar effect to pyrrocidine B in Δ*FvABC3*. Growth inhibition increased continuously until a threshold between 5 and 10 μg/mL. Pyrrocidine A + B at 10 μg/mL strongly inhibited growth, and 20 μg/mL eliminated growth. The high sensitivity of Δ*FvABC3* to pyrrocidine B, as opposed to its wild type-like pyrrocidine A response, suggests that *FvABC3* has functional specificity to pyrrocidine B.

## 4 Discussion

*Fusarium verticillioides* is a primary fungal pathogen of maize of global concern. Fumonisin contamination in maize-derived food and feed supplies threatens global food safety, human and livestock health, and international economies and trade (Nagaraj et al., 2021). As such, developing innovative solutions to mitigate fumonisin contamination in maize is critical. One such solution is the use of naturally occurring, pathosystem-relevant biological control agents to reduce *F. verticillioides* proliferation and fumonisin accumulation. *F. verticillioides* and *S. zeae* naturally co-inhabit maize kernels. *S. zeae*, as a protective endophyte of maize, is antagonistic to multiple maize kernel-rotting and mycotoxigenic fungi, including *A. flavus* and *F. verticillioides* (Wicklow and Poling, 2009). *S. zeae* and its lactam-containing secondary metabolites, the pyrrocidines, have documented success in inhibiting the growth of *F. verticillioides* and fumonisin biosynthesis (Gao et al., 2020). As such, *S. zeae* has the potential to be leveraged into a powerful biological control agent to eliminate fumonisin contamination in maize cropping systems.

Previous work by Gao et al. (2020) identified two pyrrocidine induced genes, *FvZBD1*, encoding a putative zinc-binding dehydrogenase and repressor of fumonisin biosynthesis, and *FvABC3*, encoding an ABC transporter. Gao et al. (2020) used pyrrocidine B only for further testing of mutant growth/inhibition phenotypes, assessed limited pyrrocidine doses, and did not characterize *F. verticillioides* WT growth inhibition phenotypes to varying pyrrocidine doses alongside the mutants. As previously discussed, *S. zeae* strains produce varying amounts of pyrrocidine A, B, or A + B together. Considering expectedly diverse *S. zeae* chemotypes in the field, maize kernel co-colonizing *F. verticillioides* may be presented with pyrrocidine challenges at different intensities, or a pyrrocidine A + B co-challenge. If *S. zeae* is to be deployed as a biological control agent, it is essential to know the lowest dose of pyrrocidine that can eliminate *F. verticillioides* growth, and examine the efficacy of single pyrrocidine compounds versus their combined effect. The presented research aimed to fill these gaps in the literature and represents the first study to assess (1) a comprehensive pyrrocidine A, B, and A + B (combined) dose-response assay and (2) the combined effect of pyrrocidine A + B on *F. verticillioides* growth. In addition to generating thorough information on *F. verticillioides*’ growth response to pyrrocidines, this work will help further characterize *FvZBD1* and *FvABC3* and parse out their response to, and potential role in pyrrocidine challenge.

As described, DMSO was the solvent used for all pyrrocidine compounds. DMSO is a prominent solvent used for polar and non-polar substances, and for its ability to cross hydrophobic barriers, such as cell membranes (de Abreu Costa et al., 2017). At lower concentrations, DMSO is highly useful for its capacity to increase cell permeability (by allowing pores to form) and deliver solutes intracellularly (de Abreu Costa et al., 2017). However, at higher concentrations, this same mode of action becomes cytotoxic by dissolving cell membranes. DMSO can be toxic at 10% volume for 24 h, or 5% after 120 h, suggesting that its toxicity is both concentration- and exposure time-dependent (de Abreu Costa et al., 2017). In this research, all pyrrocidine treatments, including the DMSO control, contained 0.5% DMSO. Gao et al. (2020) used up to 1% DMSO with no growth defects noted. Although our DMSO percentage (0.5%) was below the accepted toxicity levels, it was still essential to ensure that the DMSO dosage did not significantly affect *F. verticillioides* growth. To control for this potential interaction, a no-treatment control and DMSO control were used for all experiments. As expected, there was no meaningful statistical difference in total growth or final OD value (Supplementary Table S14) between the no-treatment control and 0.5% DMSO control. In wild type, the DMSO maximum growth rate was slightly less than that of the no-treatment control (Supplementary Table S15); however, this difference was not biologically relevant to the current study. These results support that *F. verticillioides* growth is not significantly affected by 0.5% DMSO dosing.

In the present study, for the first time, the combination effect of pyrrocidine A + B was analyzed. Remarkably, pyrrocidine A + B demonstrated a potent dose-dependent synergy, in which the combined compounds were more effective at inhibiting *F. verticillioides* WT growth than the individual compounds. For both individual pyrrocidine A and B compounds, inhibition of wild type growth was proportionate to pyrrocidine A and B concentration, up to the maximum concentration of 20 μg/mL. At the maximum dosage, growth was slowed but not stopped. When combined, pyrrocidine A + B inhibited wild-type cells in a typical concentration-dependent manner until a threshold was reached between 12 and 14 μg/mL, above which growth was inhibited almost entirely, and eliminated at 20 μg/mL.

The discovery of synergy between pyrrocidines A and B in inhibiting *F. verticillioides* growth is very informative for our end goal of using *S. zeae* as a biological control agent. Therefore, a maximally efficacious *S. zeae* biocontrol strain must produce both pyrrocidine A and B, potentially at a diffusible concentration of 14 μg/mL (the lowest dose tested that effectively eliminated wild type growth). This may then present the best chance of eliminating *F. verticillioides* growth and the resultant fumonisin accumulation in maize kernels in the field.

Both Δ*FvZBD1* and Δ*FvABC3* mutants exhibited increased sensitivity to pyrrocidines, in a compound-dependent manner. Δ*FvZBD1* had extreme sensitivity to pyrrocidine A (reaching a threshold effect between 5 and 10 μg/mL pyrrocidine A) and elevated sensitivity to pyrrocidine B, as compared to *F. verticillioides* WT. Δ*FvABC3* demonstrated wild type-like growth under pyrrocidine A challenge, yet had elevated sensitivity to pyrrocidine B (reaching a threshold between 10 and 20 μg/mL). Considering Δ*FvZBD1*’s severe pyrrocidine A sensitivity, and Δ*FvABC3*’s severe pyrrocidine B sensitivity, the pyrrocidine A + B combination treatment in each mutant followed that of their pyrrocidine A or B single treatment, respectively. These findings provide evidence that *FvZBD1* confers partial tolerance to pyrrocidines, and that *FvABC3*—also conferring partial tolerance to pyrrocidines—has functional specificity to pyrrocidine B. Furthermore, in wild type, pyrrocidine A had a dose-dependent higher toxicity than pyrrocidine B—as being more inhibitory to growth rate, total growth (area under the OD growth curve), and final OD value. Taken together, pyrrocidine A and B show different target specificity and synergistic action, strengthening the efficacy of an *S. zeae* biocontrol strain that produces both pyrrocidine A and B. Since pyrrocidine A and B have different targets and are synergistic, an *S. zeae* biocontrol strain that produces both pyrrocidine A and B is expected to offer stronger and more durable antimicrobial control, which should be harder for *F. verticillioides* to break or evolve resistance to (than a single compound/mode of action).

*FvZBD1* and *FvABC3* both confer partial tolerance to pyrrocidines, providing information about pyrrocidine-induced fumonisin repression, and the mechanisms of pyrrocidine tolerance in *F. verticillioides*. Fungal ABC transporters have been characterized for their critical roles in fungicide tolerance or resistance, and have been demonstrated to have substrate specificity (Sipos and Kuchler, 2006). By the likely mode of action of physical exclusion (i.e., pumping xenobiotic compounds out of the cell), *FvABC3* confers pyrrocidine B tolerance to *F. verticillioides* WT—as observed in the data, the *F. verticillioides* Δ*FvABC3* mutant was highly sensitive to pyrrocidine B specifically. *FvZBD1* encodes a putative zinc-binding dehydrogenase, with an enoyl reductase domain. Through a yet unknown mechanism, *FvZBD1* acts as a repressor of fumonisin biosynthesis and confers partial tolerance to pyrrocidines, namely pyrrocidine A. Here, we provide evidence that pyrrocidine functions through *FvZBD1* to repress fumonisin, where the Δ*FvZBD1* mutant produces fumonisins in a pyrrocidine dose-independent manner (i.e., pyrrocidine does not shut off fumonisin biosynthesis in Δ*FvZBD1* as it does in wild type).

**Figure 6:**
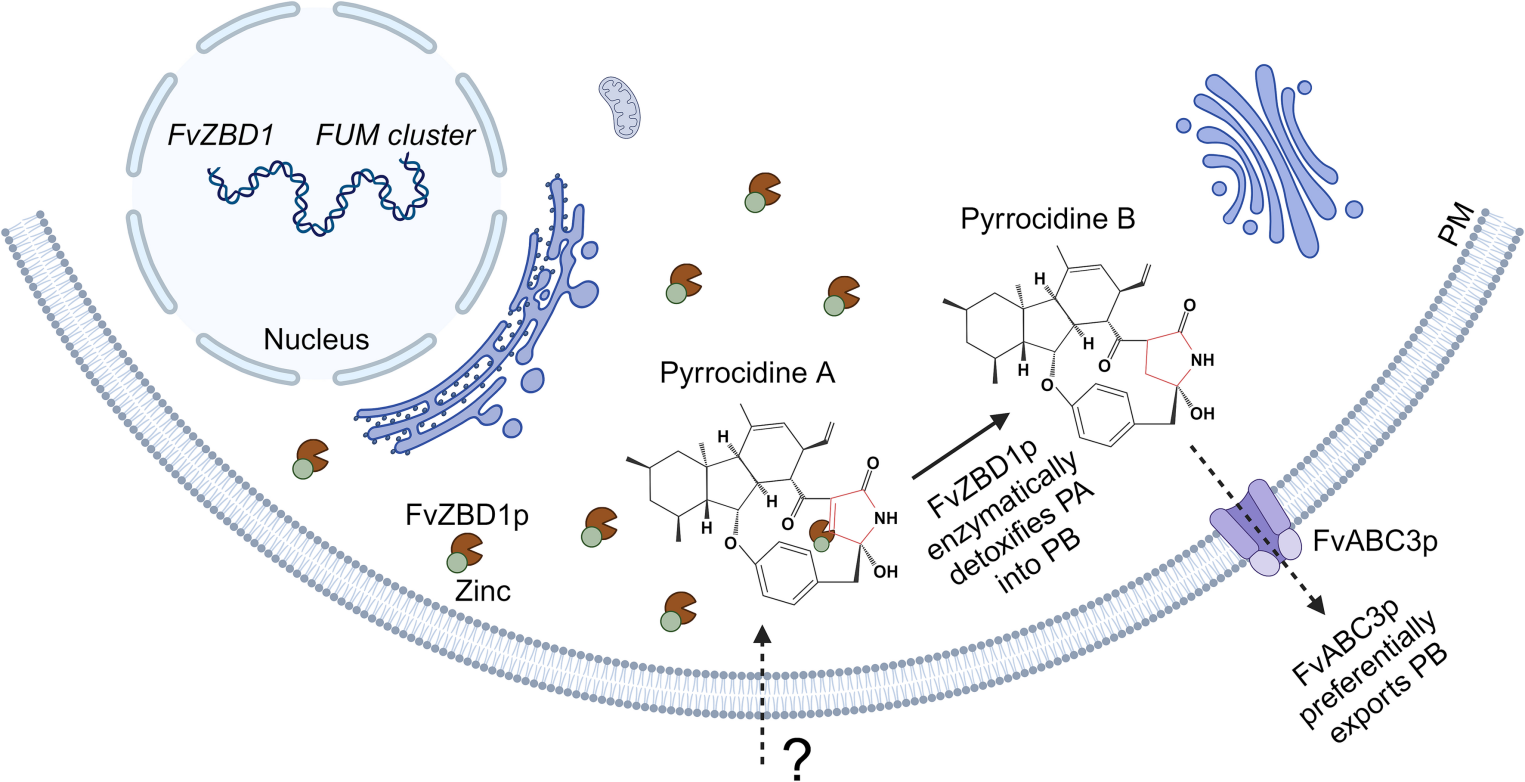
Model of *FvZBD1*- and *FvABC3*-facilitated pyrrocidine tolerance in *F. verticillioides* WT. Pyrrocidine enters the *F. verticillioides* WT cell by an unknown mechanism, inducing *FvZBD1* expression. *FvZBD1* is translated into FvZBD1p, a putative zinc-binding dehydrogenase with an enoyl reductase domain that presumably functionally requires zinc. Fumonisin biosynthesis also requires zinc (e.g., for FUM21p function—the Zn(II)2Cys6 transcription factor of the fumonisin biosynthetic gene cluster). Under pyrrocidine challenge, abundant FvZBD1p sequesters zinc, decreasing zinc availability in the cell, and thereby inhibiting fumonisin biosynthesis via competitive inhibition of FUM21p. FvZBD1p enzyme then becomes active and abundantly present in the cytoplasm. Via its enoyl reductase domain, FvZBD1p may detoxify the more toxic pyrrocidine A by reducing its double-bonded lactam ring—detoxifying pyrrocidine A into pyrrocidine B, at which FvABC3p (with specificity to pyrrocidine B) can effectively pump it out of the cell, conferring tolerance to *F. verticillioides* WT. PM, plasma membrane; PA, pyrrocidine A; PB, pyrrocidine B. Figure created with Biorender.com.

Taken together, *FvZBD1* links pyrrocidine and fumonisin into an interaction network or a unified regulatory mechanism. One model (Figure 6) of how *FvZBD1* and *FvABC3* may facilitate pyrrocidine tolerance in *F. verticillioides* WT, and antagonize fumonisin biosynthesis, is hypothesized to be the following: pyrrocidine enters the *F. verticillioides* cell (by a yet unknown mechanism), and pyrrocidine challenge highly induces *FvZBD1* expression. *FvZBD1* is abundantly translated into FvZBD1p, a putative zinc-binding dehydrogenase. FvZBD1p presumably requires zinc. Fumonisin biosynthesis also requires zinc. For example, zinc is required by FUM21p (FVEG_00315), the Zn(II)2Cys6 transcription factor of the fumonisin biosynthetic gene cluster. Under pyrrocidine challenge, abundant FvZBD1p sequesters zinc, and decreases zinc availability in the cell, thereby inhibiting fumonisin biosynthesis via competitive inhibition of FUM21p (substrate sequestration). FvZBD1p enzyme is then active and abundantly present in the cytoplasm. Via its enoyl reductase domain, FvZBD1p may detoxify pyrrocidine A (by reducing its double-bonded lactam ring) into pyrrocidine B, at which point FvABC3p can then effectively pump pyrrocidine B out of the cell, conferring tolerance to *F. verticillioides* WT.

In support of this model, pyrrocidine A and B show different target specificity (*FvZBD1* and *FvABC3*, respectively) and synergistic activity. Δ*FvZBD1* was more sensitive to pyrrocidine A, which, following the model would be an expected phenotype if *FvZBD1* were required for pyrrocidine A detoxification. Further, Δ*FvABC3* was more sensitive to pyrrocidine B, which would also be an expected phenotype if *FvABC3* had functional specificity to pyrrocidine B and were required for pumping pyrrocidine B out of the cell. *FvZBD1* has a possible enoyl reductase domain, which is hypothesized here to enzymatically degrade pyrrocidine A into B. In the pyrrocidine biosynthetic pathway of *S. zeae*, an enoyl reductase is responsible for reducing pyrrocidine A into pyrrocidine B (Chen et al., 2024), supporting that an enoyl reductase has the capability to perform this reaction. As such, it is then possible that FvZBD1p’s enoyl reductase domain may perform this same function in *F. verticillioides*. Reduced zinc availability in the cell (by FvZBD1p zinc sequestration) would hinder many cellular process, which may be represented here as general growth inhibition.

To test the model, future planned work will determine the activity of FvZBD1p on pyrrocidine A via experiments using cell-free extracts, or directly with enzymatic assays. Preliminary metabolomics data, assessing pyrrocidine B production from pyrrocidine A treated *F. verticillioides* WT or Δ*FvZBD1* cells, supports that pyrrocidine B is made by wild type in response to pyrrocidine A challenge. To test if FvZBD1p sequesters zinc in the cell under pyrrocidine challenge, intracellular zinc availability could be measured by sensors [e.g., small molecule probes or Förster Resonance Energy Transfer-based sensors (Carpenter et al., 2016)]. Alternatively, zinc could be supplemented to the *F. verticillioides*-pyrrocidine interaction. If zinc supplementation rescued fumonisin production (e.g., by allowing FUM21p function), the hypothesis would be supported. This model proposes that pyrrocidine enters the cell naturally *in planta*, by a yet unknown mechanism. This presumed natural mechanism (e.g., an influx protein) requires further exploration. Future work will employ untargeted metabolomics and other multi-omic methodologies to further elucidate the molecular mechanisms driving pyrrocidine-induced *FvZBD1* expression and pyrrocidine- and FvZBD1p-repressed fumonisin production, and investigate the efficacy of pyrrocidine-based fumonisin inhibition.

In summary, this research represents the first report that pyrrocidines A and B act powerfully and synergistically to inhibit *F. verticillioides* growth, and reveals that pyrrocidine A and B have different target activities. Further, we provide evidence that *FvZBD1* confers partial tolerance to pyrrocidine, and that pyrrocidine functions through *FvZBD1* to repress fumonisin biosynthesis; however, the function of *FvZBD1* remains unconfirmed. Pyrrocidine dose-response studies also identified that *FvABC3* has functional specificity to pyrrocidine B. The presented functional model proposes a highly specific/stringent co-evolution system, in which *F. verticillioides*’ *FvZBD1* has co-evolved to detoxify *S. zeae*’s antimicrobial pyrrocidine A into pyrrocidine B, which can be effectively eliminated via the *FvABC3* export pump—conferring tolerance, higher fitness, and sustained competition in the maize kernel environment. This novel study advances our knowledge of competitive fungal relationships occurring in the maize kernel and the role of secondary metabolites in fungal communication and mycotoxin control. Further, the discovery of synergy between the naturally occurring pyrrocidine A and B compounds, highlighting their combined efficacy, reminds us of the power of examining biological interactions as a complete system, rather than as isolated facets. This and future untargeted metabolomic work will inform the identification and/or development of powerful biological control strains of *S. zeae*—that produce both pyrrocidines A and B, in an optimized ratio—leveraged to eliminate fumonisin contamination in maize across conventional, organic, and subsistence agriculture.

## Data Availability

The datasets presented in this study can be found in online repositories. The names of the repository/repositories and accession number(s) can be found below: all original data and code required to reproduce the analyses are publicly available in the USDA Ag Data Commons (DOI: 10.15482/USDA.ADC/26239976; Lofton et al. 2024).
