# Supplementary material for: Pyrrocidines A and B demonstrate synergistic inhibition of Fusarium verticillioides growth
Source: Front Microbiol. 2025 Jan 9;15:1480920. doi: 10.3389/fmicb.2024.1480920 (PMC11754276; doi:10.3389/fmicb.2024.1480920)
Supplement: Supplementary file 1 [file Data_Sheet_1.docx]

Supplementary Material

# Supplementary Figures and Tables

## Supplementary Figures


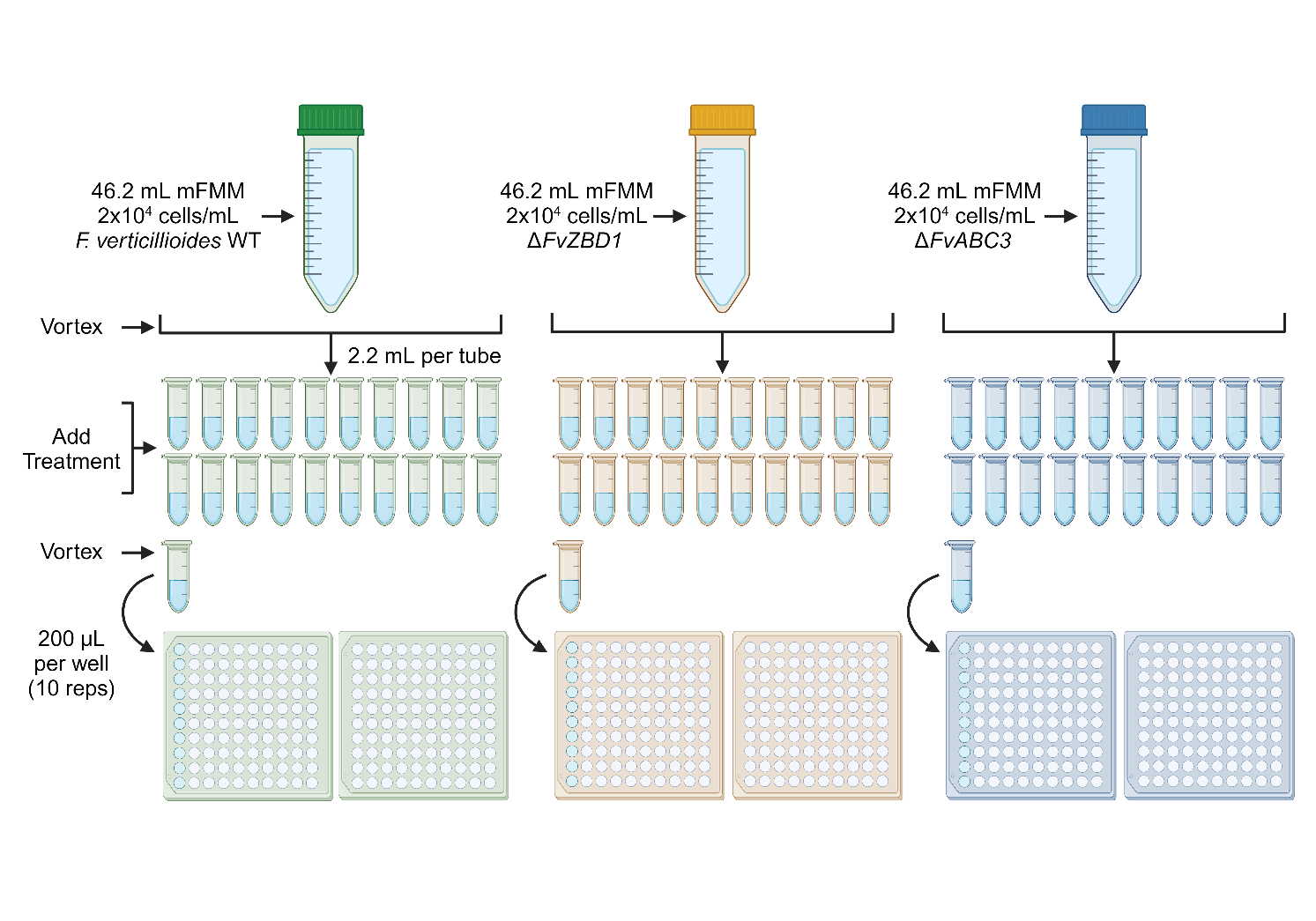


**Supplementary Figure 1.** **Experimental workflow for the *F. verticillioides* pyrrocidine dose-response assay.** First, a master mix of *F. verticillioides* conidia and growth media was prepared per strain, at 2×10^4^ conidia/mL modified *Fusarium* minimal media (mFMM). Second, stocks were prepared per pyrrocidine compound and treatment dose. Treatment stocks were combined with the cells and media to generate a master mix of the appropriate treatment conditions. Each treatment condition master mix was then distributed into Bioscreen honeycomb microtiter plates, with 10 technical replicates per treatment condition. Microtiter plates were incubated for 5 days (120 hours) in the dark, at 28°C, with continuous shaking. Fungal growth measurements (as OD A600nm) were recorded every 30 minutes. mFMM = modified *Fusarium* minimal media. WT = wild type. Figure created with Biorender.com.


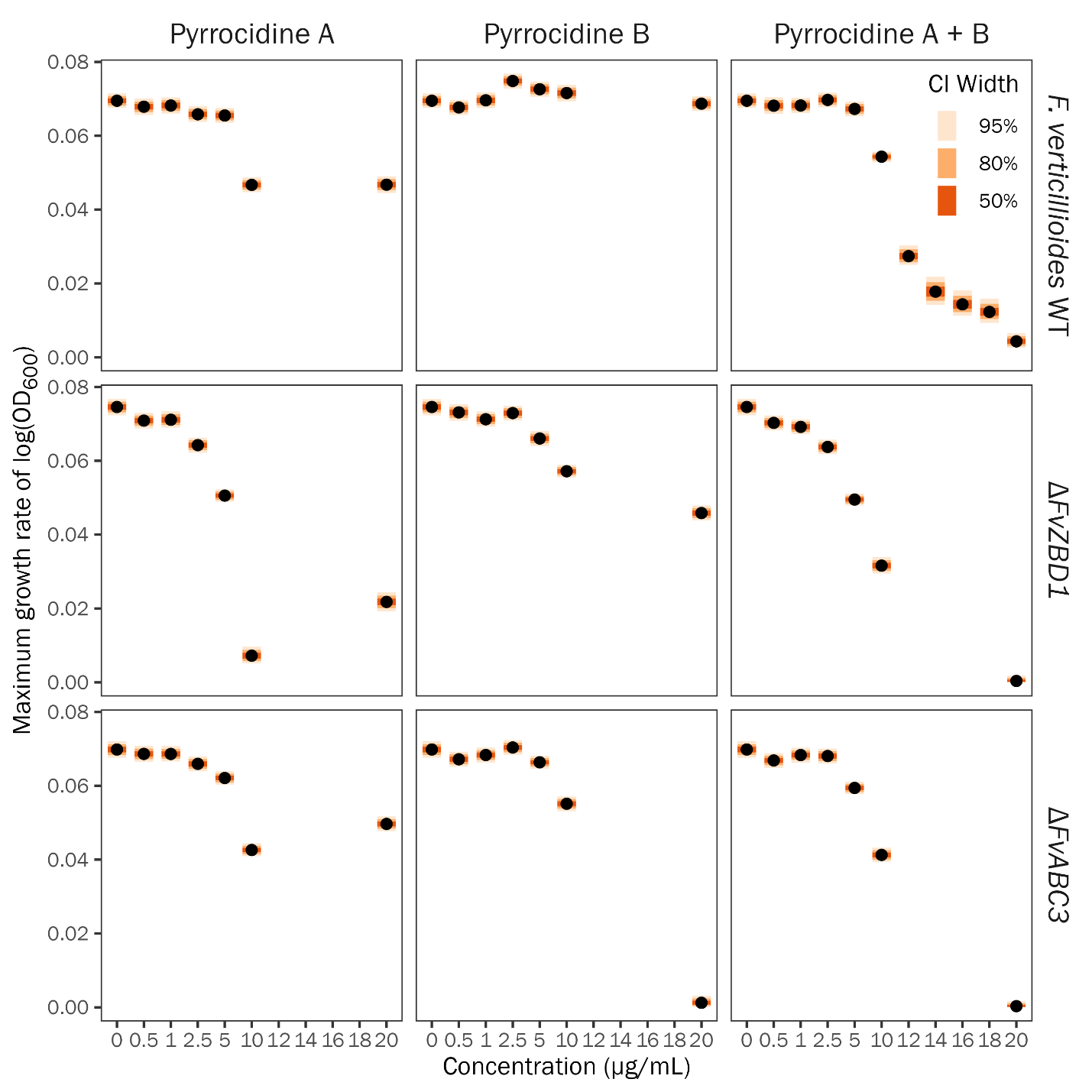


**Supplementary Figure 2.** **Pyrrocidine A, B, and combination A+B inhibit *F. verticillioides* growth rate in a dose-, compound-, and strain-dependent manner.** Plot of the fitted model values for the maximum growth rate of *F. verticillioides* WT, Δ*FvZBD1*, and Δ*FvABC3* strains challenged with the following treatment conditions: 0.5% DMSO control; 0.5% DMSO containing either pyrrocidine A, B, or combination A+B at the following final concentrations, (1) 0.5, (2) 1.0, (3) 2.5, (4) 5.0, (5) 10, and (6) 20 μg/mL, monitored for 120 hours, at 28°C, with continuous shaking in the dark in modified *Fusarium* minimal media. Ten replicates were tested per treatment, and optical density (OD600) measurements were recorded every 30 minutes. Medians of the posterior estimates of the maximum growth rate are shown as points and credible intervals as progressively lighter-shaded bars. CI = credible interval.


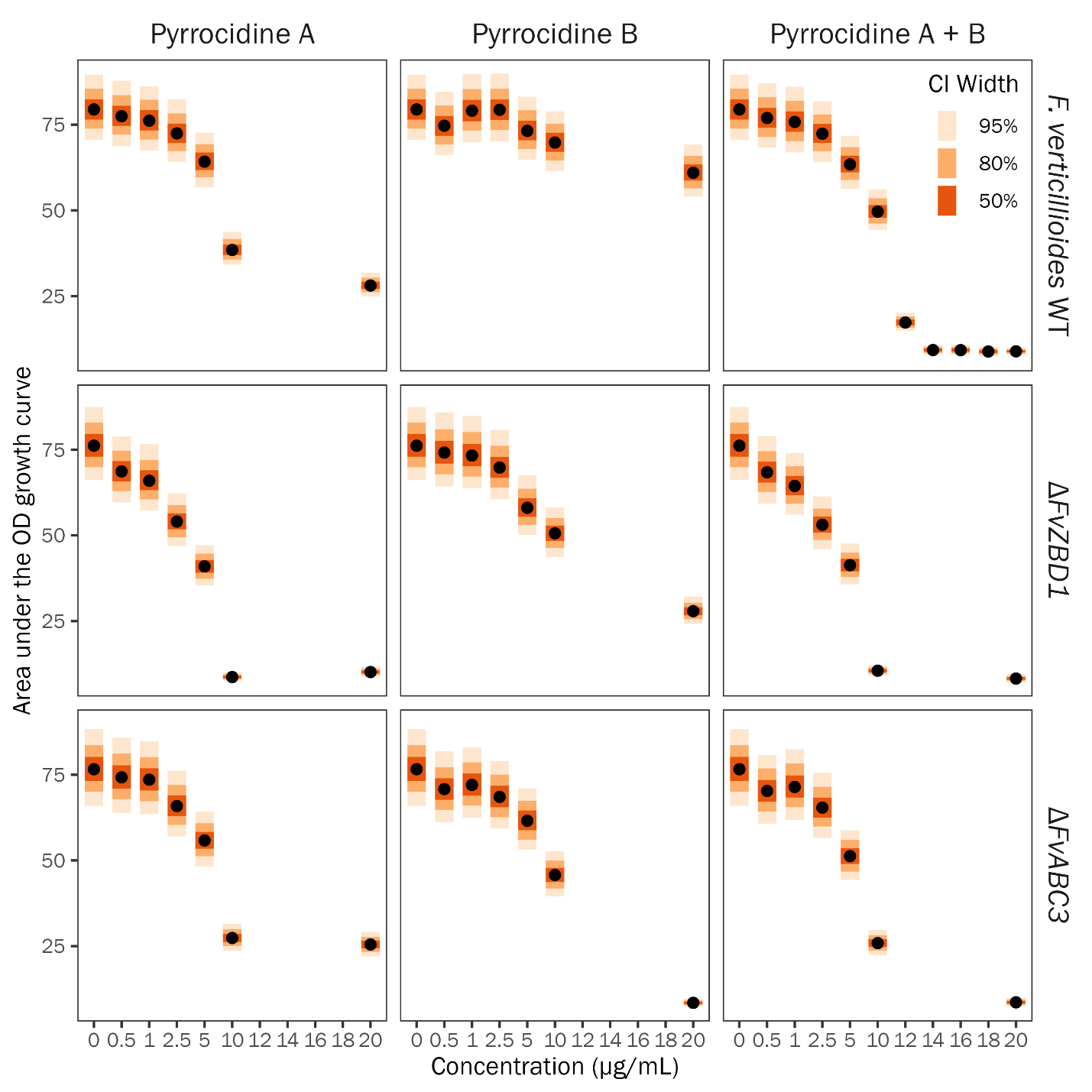


**Supplementary Figure 3. Pyrrocidine A, B, and combination A+B inhibit *F. verticillioides* total growth in a dose-, compound-, and strain-dependent manner.** Plot of the fitted model values for the total growth (area under the optical density (OD) growth curve) of *F. verticillioides* WT, Δ*FvZBD1*, and Δ*FvABC3* strains challenged with the following treatment conditions: 0.5% DMSO control; 0.5% DMSO containing either pyrrocidine A, B, or combination A+B at the following final concentrations, (1) 0.5, (2) 1.0, (3) 2.5, (4) 5.0, (5) 10, and (6) 20 μg/mL, monitored for 120 hours, at 28°C, with continuous shaking in the dark in modified *Fusarium* minimal media. Ten replicates were tested per treatment, and optical density (OD600) measurements were recorded every 30 minutes. Medians of the posterior estimates of total growth are shown as points and credible intervals as progressively lighter-shaded bars. CI = credible interval.


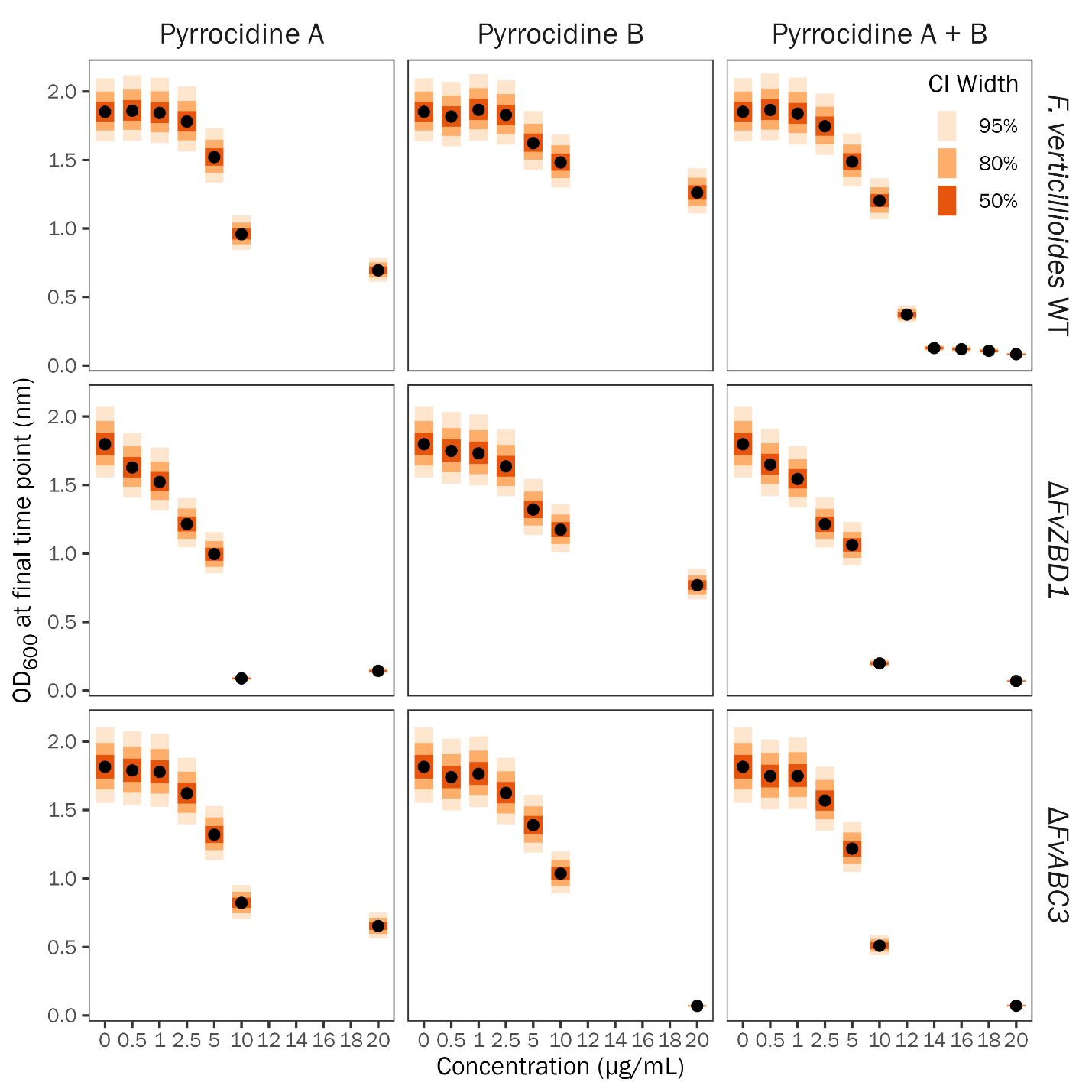


**Supplementary Figure 4. Pyrrocidine A, B, and combination A+B inhibit the *F. verticillioides* final OD value in a dose-, compound-, and strain-dependent manner.** Plot of the fitted model values for the optical density (OD) at the final time point of *F. verticillioides* WT, Δ*FvZBD1*, and Δ*FvABC3* strains challenged with the following treatment conditions: 0.5% DMSO control; 0.5% DMSO containing either pyrrocidine A, B, or combination A+B at the following final concentrations, (1) 0.5, (2) 1.0, (3) 2.5, (4) 5.0, (5) 10, and (6) 20 μg/mL, monitored for 120 hours, at 28°C, with continuous shaking in the dark in modified *Fusarium* minimal media. Ten replicates were tested per treatment, and optical density (OD600) measurements were recorded every 30 minutes. Medians of the posterior estimates of the final OD are shown as points and credible intervals as progressively lighter-shaded bars. CI = credible interval.


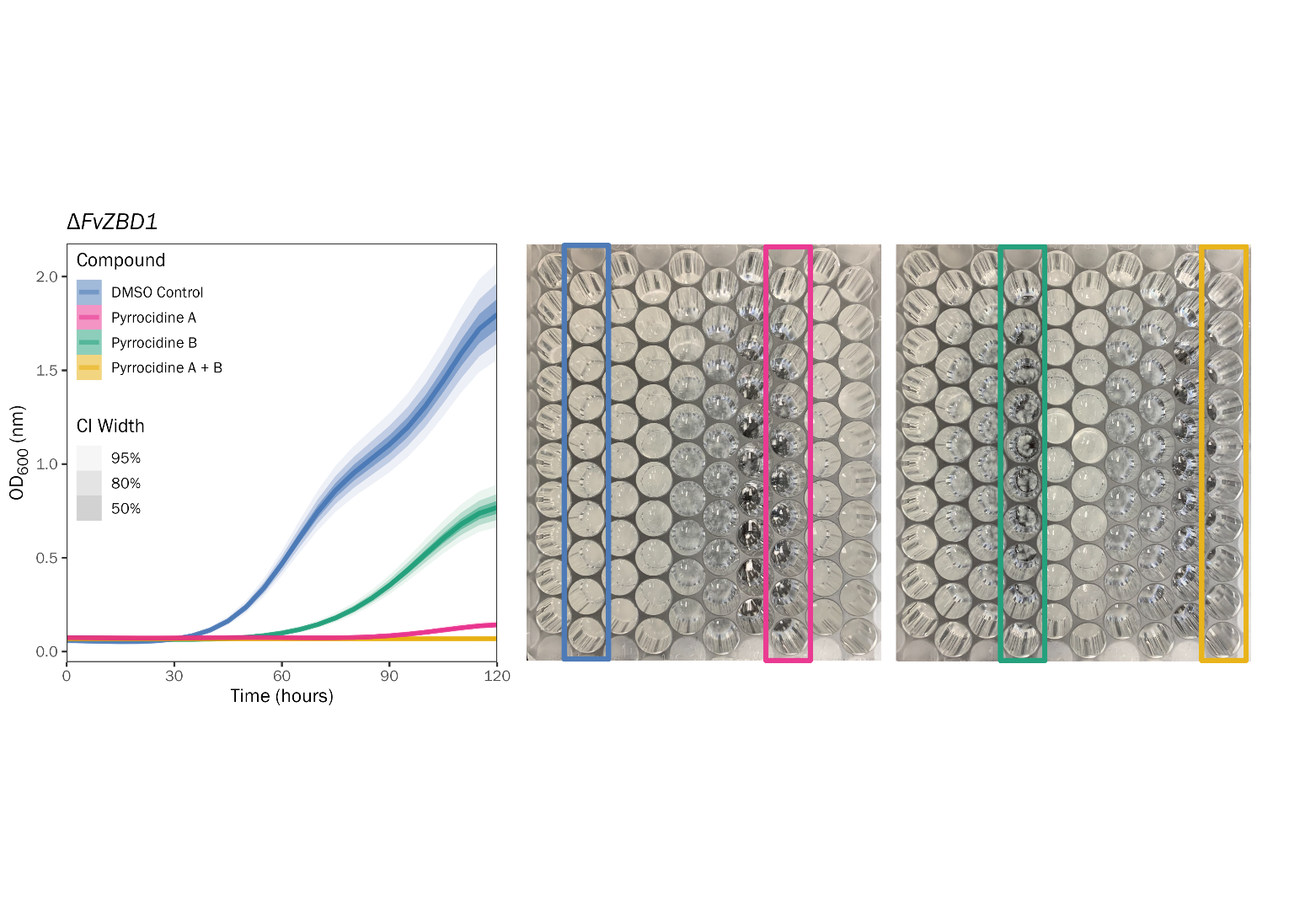


**Supplementary Figure 5. Δ*FvZBD1* presented a stress-induced irregular growth phenotype in response to pyrrocidine B challenge.** Growth curve analysis of the *F. verticillioides* Δ*FvZBD1* mutant challenged with the following treatment conditions: 0.5% DMSO control; 0.5% DMSO containing either pyrrocidine A, B, or combination A+B at the final concentration of 20 μg/mL, monitored for 120 hours, at 28°C, with continuous shaking in the dark in modified *Fusarium* minimal media. Ten replicates were tested per treatment, and optical density (OD600) measurements were recorded every 30 minutes. Plot of the fitted model values mapped to the corresponding growth inhibition phenotypes, highlighted in the microtiter plates after 120-hour incubation. Medians of the posterior distributions are plotted as thick lines, with credible intervals shown as progressively lighter-shaded areas. CI = credible interval. Figure created with Biorender.com.


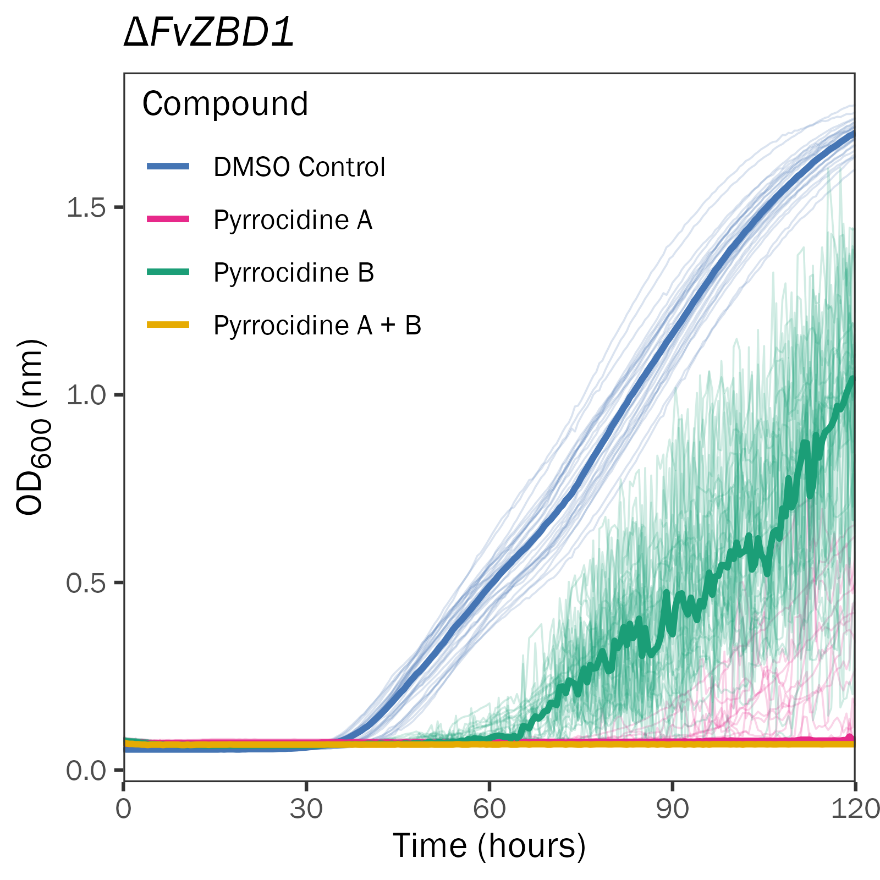


**Supplementary Figure 6.** ***FvZBD1* confers partial tolerance to pyrrocidines.** Growth curve analysis of the *F. verticillioides* Δ*FvZBD1* mutant challenged with the following treatment conditions: 0.5% DMSO control; 0.5% DMSO containing either pyrrocidine A, B, or combination A+B at the final concentration of 20 μg/mL, monitored for 120 hours, at 28°C, with continuous shaking in the dark in modified *Fusarium* minimal media. Ten replicates were tested per treatment, and optical density (OD600) measurements were recorded every 30 minutes. Plot of the raw data. Individual wells (replications) are plotted as thin semi-transparent lines, and medians are plotted as thick lines.


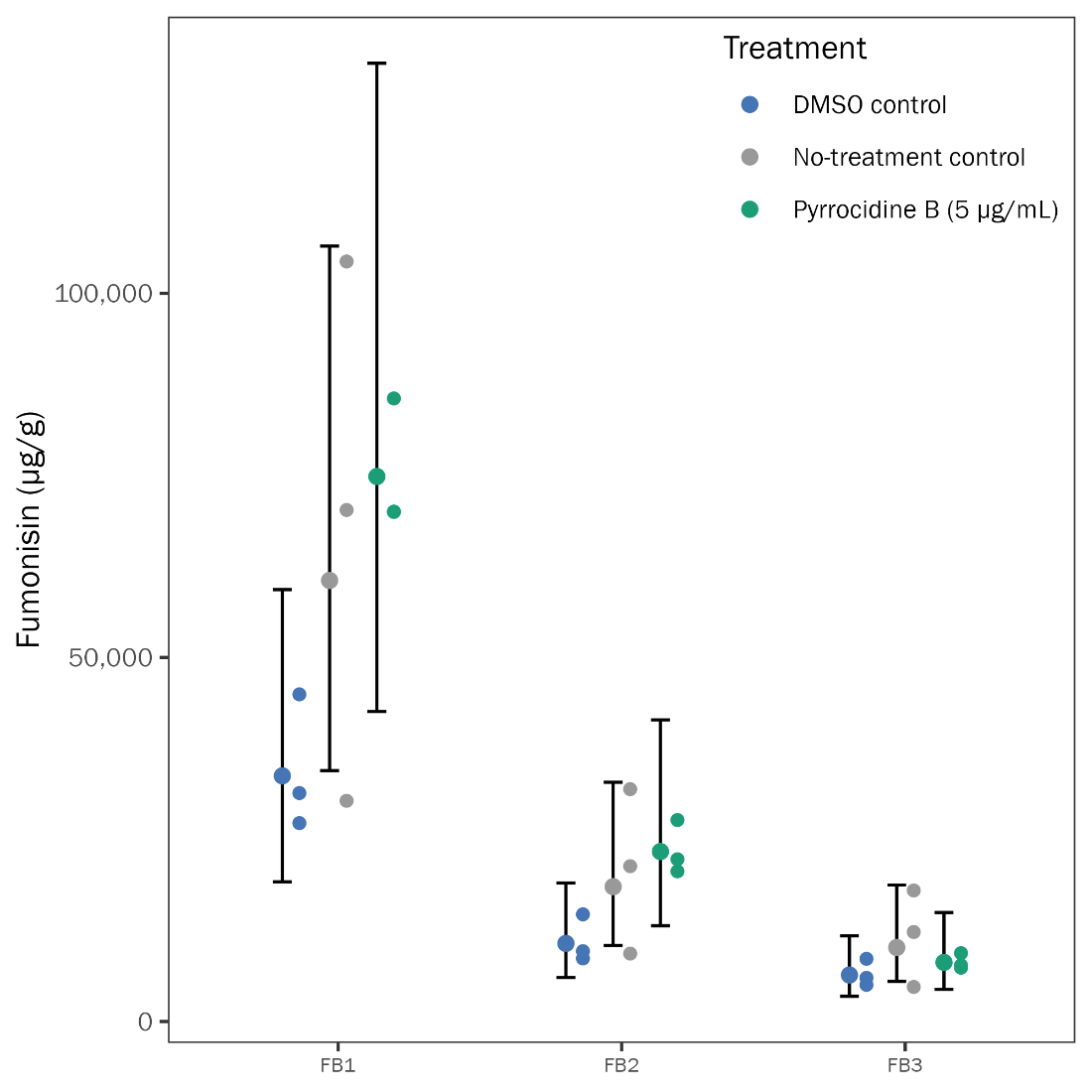


**Supplementary Figure 7. *F. verticillioides*** **Δ*FvZBD1* mutant fumonisin biosynthesis was not inhibited by 5 μg/mL pyrrocidine B challenge.** 3 mL PDB starter cultures of *F. verticillioides* WT (FRC M-3125) and the Δ*FvZBD1* (Δ*FvZBD1*-1; FVEG_00314-4-1) deletion mutant (Gao et al., 2020) were grown in a dark incubator, at 27°C, 250 rpm, for 3 days. 10 µL of each starter culture (per strain) was then inoculated into 9 tubes (14 mL, sterile, round bottom, snap cap tubes) containing 3 mL fresh PDB. Tubes were incubated with the caps unsnapped in a dark incubator, at 27°C, 250 rpm, for 24 hours – after which the following treatments were added: no-treatment control; 0.5% DMSO control; or 0.5% DMSO containing pyrrocidine B at a final concentration of 5 μg/mL. Three technical replicates were tested per treatment. Tubes were incubated as above for an additional 4 days post-treatment, then processed and analyzed for fumonisin (FB_1_, FB_2_, FB_3_) content as described in the *Fumonisin Quantification* Materials and Methods section (2.5). Similar statistical models as described in the *Statistical* *Analysis of ΔFvZBD1 Fumonisin Data* Materials and Methods section (2.7) were fit to these data, with only three discrete treatment levels (no-treatment control, DMSO control, and pyrrocidine B at 5 µg/mL). The graph shows the distribution of the raw data (points) and the means with 95% confidence intervals for FB_1_, FB_2_, and FB_3_ – indicating no significant difference in mean fumonisin biosynthesis between the three treatments.

## Supplementary Tables

**Supplementary Table 1.** *F. verticillioides* pyrrocidine dose-response assay, treatment conditions per genotype.

| Treatment Conditions | | | | | | |
| --- | --- | --- | --- | --- | --- | --- |
| Controls | No-treatment control | 0.5% DMSO control |  | | | |
| Pyrrocidine A | 0.5 μg/mL pyrrocidine A | 1.0 μg/mL pyrrocidine A | 2.5 μg/mL pyrrocidine A | 5.0 μg/mL pyrrocidine A | 10 μg/mL pyrrocidine A | 20 μg/mL pyrrocidine A |
| Pyrrocidine B | 0.5 μg/mL pyrrocidine B | 1.0 μg/mL pyrrocidine B | 2.5 μg/mL pyrrocidine B | 5.0 μg/mL pyrrocidine B | 10 μg/mL pyrrocidine B | 20 μg/mL pyrrocidine B |
| Pyrrocidine A+B | 0.5 μg/mL pyrrocidine A+B  (i.e., 0.25 μg/mL pyrrocidine A + 0.25 μg/mL pyrrocidine B) | 1.0 μg/mL pyrrocidine A+B  (i.e., 0.5 μg/mL pyrrocidine A + 0.5 μg/mL pyrrocidine B) | 2.5 μg/mL pyrrocidine A+B  (i.e., 1.25 μg/mL pyrrocidine A + 1.25 μg/mL pyrrocidine B) | 5.0 μg/mL pyrrocidine A+B  (i.e., 2.5 μg/mL pyrrocidine A + 2.5 μg/mL pyrrocidine B) | 10 μg/mL pyrrocidine A+B  (i.e., 5 μg/mL pyrrocidine A +  5 μg/mL pyrrocidine B) | 20 μg/mL pyrrocidine A+B  (i.e., 10 μg/mL pyrrocidine A + 10 μg/mL pyrrocidine B) |

**Supplementary Table 2.** Treatment conditions for the supplementary pyrrocidine dose-response assay to assess the treatment effect of pyrrocidine A+B in combination between 10 and 20 μg/mL on *F. verticillioides* WT.

| Treatment Conditions | | | | | | |
| --- | --- | --- | --- | --- | --- | --- |
| Controls | No-treatment control | 0.5% DMSO control |  | | | |
| Pyrrocidine A+B | 10 μg/mL pyrrocidine A+B  (i.e., 5 μg/mL pyrrocidine A +  5 μg/mL pyrrocidine B) | 12 μg/mL pyrrocidine A+B  (i.e., 6 μg/mL pyrrocidine A +  6 μg/mL pyrrocidine B) | 14 μg/mL pyrrocidine A+B  (i.e., 7 μg/mL pyrrocidine A +  7 μg/mL pyrrocidine B) | 16 μg/mL pyrrocidine A+B  (i.e., 8 μg/mL pyrrocidine A +  8 μg/mL pyrrocidine B) | 18 μg/mL pyrrocidine A+B  (i.e., 9 μg/mL pyrrocidine A +  9 μg/mL pyrrocidine B) | 20 μg/mL pyrrocidine A+B  (i.e., 10 μg/mL pyrrocidine A + 10 μg/mL pyrrocidine B) |

**Supplementary Table 3. 0.5% DMSO does not significantly affect *F. verticillioides* total growth.** Comparisons of fitted model values of total growth (area under the optical density (OD) growth curve) of the *F. verticillioides* WT, Δ*FvZBD1*, and Δ*FvABC3* no-treatment control and 0.5% DMSO control, within each strain. Based on the large 95% credible intervals and >0.05 p_MAP_ values, there was no meaningful statistical difference in total growth between the no-treatment control and DMSO control for the *F. verticillioides* WT, Δ*FvZBD1*, or Δ*FvABC3* strains.

| Strain | Difference Between Total Growth in No-Treatment Control and 0.5% DMSO Control | 95% Credible Interval | p_MAP_ |
| --- | --- | --- | --- |
| *F. verticillioides* WT | 6.335 | (0.76, 12.238) | 0.094 |
| Δ*FvZBD1* | 1.983 | (-4.204, 8.606) | 0.885 |
| Δ*FvABC3* | 3.826 | (-2.458, 10.211) | 0.556 |

**Supplementary Table 4. Pyrrocidine A demonstrated a dose-dependent higher toxicity than pyrrocidine B with increased inhibition of *F. verticillioides* WT growth rate.** Comparisons of fitted model values by compound:concentration combination, of the maximum growth rate of *F. verticillioides* WT challenged with pyrrocidine A or B at the following final concentrations: (1) 0.5, (2) 1.0, (3) 2.5, (4) 5.0, (5) 10, and (6) 20 μg/mL. As shown in <0.05 p_MAP_ values, pyrrocidine A inhibited the maximum growth rate of *F. verticillioides* WT significantly more than pyrrocidine B in the 2.5 μg/mL and higher doses. Higher growth inhibition is interpreted as higher toxicity.

| Treatment Combination 1 | Treatment Combination 2 | Difference Between Maximum Growth Rates | 95% Credible Interval | p_MAP_ |
| --- | --- | --- | --- | --- |
| Pyrrocidine A:0.5 μg/mL | Pyrrocidine B:0.5 μg/mL | 0.000 | (-0.003, 0.003) | 0.996 |
| Pyrrocidine A:1 μg/mL | Pyrrocidine B:1 μg/mL | −0.001 | (-0.005, 0.002) | 0.670 |
| Pyrrocidine A:2.5 μg/mL | Pyrrocidine B:2.5 μg/mL | −0.009 | (-0.012, -0.006) | < 0.001 |
| Pyrrocidine A:5 μg/mL | Pyrrocidine B:5 μg/mL | −0.007 | (-0.01, -0.004) | < 0.001 |
| Pyrrocidine A:10 μg/mL | Pyrrocidine B:10 μg/mL | −0.025 | (-0.028, -0.022) | < 0.001 |
| Pyrrocidine A:20 μg/mL | Pyrrocidine B:20 μg/mL | −0.022 | (-0.025, -0.019) | < 0.001 |

**Supplementary Table 5. Pyrrocidine A demonstrated a dose-dependent higher toxicity than pyrrocidine B with increased inhibition of *F. verticillioides* WT total growth.** Comparisons of fitted model values by compound:concentration combination, of the total growth (area under the optical density (OD) growth curve) of *F. verticillioides* WT, challenged with pyrrocidine A or B at the following final concentrations: (1) 0.5, (2) 1.0, (3) 2.5, (4) 5.0, (5) 10, and (6) 20 μg/mL. As shown in <0.05 p_MAP_ values, pyrrocidine A inhibited the total growth of *F. verticillioides* WT significantly more than pyrrocidine B in the 5 μg/mL and higher doses. Higher growth inhibition is interpreted as higher toxicity.

| Treatment Combination 1 | Treatment Combination 2 | Difference Between Total Growth | 95% Credible Interval | p_MAP_ |
| --- | --- | --- | --- | --- |
| Pyrrocidine A:0.5 μg/mL | Pyrrocidine B:0.5 μg/mL | 2.820 | (-3.471, 9.434) | 0.675 |
| Pyrrocidine A:1 μg/mL | Pyrrocidine B:1 μg/mL | −2.904 | (-9.229, 3.517) | 0.669 |
| Pyrrocidine A:2.5 μg/mL | Pyrrocidine B:2.5 μg/mL | −6.771 | (-12.935, -0.881) | 0.094 |
| Pyrrocidine A:5 μg/mL | Pyrrocidine B:5 μg/mL | −8.949 | (-15.078, -3.439) | 0.007 |
| Pyrrocidine A:10 μg/mL | Pyrrocidine B:10 μg/mL | −31.318 | (-37.297, -25.515) | < 0.001 |
| Pyrrocidine A:20 μg/mL | Pyrrocidine B:20 μg/mL | −32.866 | (-38.642, -27.887) | < 0.001 |

**Supplementary Table 6. Pyrrocidine A demonstrated a dose-dependent higher toxicity than pyrrocidine B with increased inhibition of the *F. verticillioides* WT final OD value.** Comparisons of fitted model values by compound:concentration combination, of the optical density (OD) at the final time point of *F. verticillioides* WT, challenged with pyrrocidine A or B at the following final concentrations: (1) 0.5, (2) 1.0, (3) 2.5, (4) 5.0, (5) 10, and (6) 20 μg/mL. As shown in <0.05 p_MAP_ values, pyrrocidine A inhibited the final OD of *F. verticillioides* WT significantly more than pyrrocidine B in the 10 μg/mL and higher doses. Higher growth inhibition is interpreted as higher toxicity.

| Treatment Combination 1 | Treatment Combination 2 | Difference Between Final OD | 95% Credible Interval | p_MAP_ |
| --- | --- | --- | --- | --- |
| Pyrrocidine A:0.5 μg/mL | Pyrrocidine B:0.5 μg/mL | 0.043 | (-0.139, 0.235) | 0.827 |
| Pyrrocidine A:1 μg/mL | Pyrrocidine B:1 μg/mL | −0.023 | (-0.209, 0.161) | 0.969 |
| Pyrrocidine A:2.5 μg/mL | Pyrrocidine B:2.5 μg/mL | −0.047 | (-0.226, 0.126) | 0.896 |
| Pyrrocidine A:5 μg/mL | Pyrrocidine B:5 μg/mL | −0.104 | (-0.268, 0.048) | 0.415 |
| Pyrrocidine A:10 μg/mL | Pyrrocidine B:10 μg/mL | −0.524 | (-0.668, -0.387) | < 0.001 |
| Pyrrocidine A:20 μg/mL | Pyrrocidine B:20 μg/mL | −0.567 | (-0.695, -0.455) | < 0.001 |

**Supplementary Table 7. Pyrrocidines A and B act synergistically to inhibit *F. verticillioides* WT growth.** Comparisons of fitted model values of the synergistic effect of 20 μg/mL pyrrocidine A+B versus the additive effect of 10 μg/mL pyrrocidine A and B for *F. verticillioides* WT. The "synergistic effect" is the mean of the growth parameter in the DMSO control minus the mean of the parameter in the 20 μg/mL pyrrocidine A+B treatment. The "sum of additive effects" is the sum of two differences: the difference between the DMSO control mean and the 10 μg/mL pyrrocidine A mean; and the difference between the DMSO control mean and the 10 μg/mL pyrrocidine B mean. The "synergistic:additive ratio" is the number of times greater the synergistic effect is compared to the additive effect. The p_MAP_ value is against the null hypothesis that this ratio is equal to 1. 95% credible intervals for all quantities are given in parentheses. In each parameter (growth rate, total growth, or final optical density (OD)), the synergistic effect is significantly higher than that of the additive effect, meaning that pyrrocidine A and B act synergistically (not additively) to inhibit *F. verticillioides* WT growth.

| Growth Parameter | Synergistic Effect | Sum of Additive Effects | Synergistic:Additive Ratio | p_MAP_ |
| --- | --- | --- | --- | --- |
| Maximum Growth Rate | 0.07 (0.06, 0.07) | 0.02 (0.02, 0.03) | 3.15 (2.59, 4.06) | < 0.0001 |
| Total Growth  (area under the OD growth curve) | 70.55 (62.57, 79.88) | 50.62 (40.47, 61.62) | 1.39 (1.23, 1.63) | < 0.0001 |
| OD at the Final Time Point | 1.77 (1.56, 2.00) | 1.26 (0.99, 1.56) | 1.40 (1.23, 1.67) | < 0.0001 |

**Supplementary Table 8. The *F. verticillioides* Δ*FvZBD1* mutant exhibited extreme sensitivity to pyrrocidine A and elevated sensitivity to pyrrocidine B as demonstrated by total growth inhibition.** Comparisons of fitted model values by strain within concentration and compound, of the total growth (area under the optical density (OD) growth curve) of *F. verticillioides* WT and Δ*FvZBD1* mutant challenged with pyrrocidine A, B, or combination A+B at the following final concentrations: (1) 0.5, (2) 1.0, (3) 2.5, (4) 5.0, (5) 10, and (6) 20 μg/mL. As shown in narrower 95% credible intervals and <0.05 p_MAP_ values, Δ*FvZBD1* exhibited significantly less total growth under 2.5 μg/mL pyrrocidine A challenge and higher, 10 μg/mL pyrrocidine B challenge and higher, and 2.5 μg/mL pyrrocidine A+B combined challenge and higher. See **Supplementary** **Tables 9** and **10** for comparisons of the maximum growth rate and final OD value. *To note, the 20 μg/mL pyrrocidine A+B treatment is not statistically meaningful because growth was completely inhibited for both wild type and Δ*FvZBD1* under this treatment condition.

| Compound | Concentration | Strain 1 | Strain 2 | Difference Between Total Growth | 95% Credible Interval | p_MAP_ |
| --- | --- | --- | --- | --- | --- | --- |
| Pyrrocidine A | 0.5 μg/mL | *F. verticillioides* WT | Δ*FvZBD1* | 8.922 | (-5.085, 22.218) | 0.370 |
| Pyrrocidine A | 1 μg/mL | *F. verticillioides* WT | Δ*FvZBD1* | 10.267 | (-3.74, 23.279) | 0.247 |
| Pyrrocidine A | 2.5 μg/mL | *F. verticillioides* WT | Δ*FvZBD1* | 18.362 | (6.649, 30.727) | 0.013 |
| Pyrrocidine A | 5 μg/mL | *F. verticillioides* WT | Δ*FvZBD1* | 23.326 | (13.598, 33.215) | 0.003 |
| Pyrrocidine A | 10 μg/mL | *F. verticillioides* WT | Δ*FvZBD1* | 29.798 | (25.163, 35.08) | < 0.001 |
| Pyrrocidine A | 20 μg/mL | *F. verticillioides* WT | Δ*FvZBD1* | 17.913 | (14.375, 21.922) | < 0.001 |
| Pyrrocidine B | 0.5 μg/mL | *F. verticillioides* WT | Δ*FvZBD1* | 0.457 | (-14.37, 14.035) | 0.999 |
| Pyrrocidine B | 1 μg/mL | *F. verticillioides* WT | Δ*FvZBD1* | 5.672 | (-9.24, 19.74) | 0.617 |
| Pyrrocidine B | 2.5 μg/mL | *F. verticillioides* WT | Δ*FvZBD1* | 9.401 | (-4.619, 23.355) | 0.326 |
| Pyrrocidine B | 5 μg/mL | *F. verticillioides* WT | Δ*FvZBD1* | 15.167 | (2.561, 27.508) | 0.051 |
| Pyrrocidine B | 10 μg/mL | *F. verticillioides* WT | Δ*FvZBD1* | 19.216 | (7.653, 30.679) | 0.010 |
| Pyrrocidine B | 20 μg/mL | *F. verticillioides* WT | Δ*FvZBD1* | 33.099 | (24.92, 41.883) | < 0.001 |
| Pyrrocidine A+B | 0.5 μg/mL  (i.e., 0.25 μg/mL pyrrocidine A + 0.25 μg/mL pyrrocidine B) | *F. verticillioides* WT | Δ*FvZBD1* | 8.541 | (-5.11, 21.865) | 0.363 |
| Pyrrocidine A+B | 1 μg/mL  (i.e., 0.5 μg/mL pyrrocidine A + 0.5 μg/mL pyrrocidine B) | *F. verticillioides* WT | Δ*FvZBD1* | 11.366 | (-2.319, 24.284) | 0.181 |
| Pyrrocidine A+B | 2.5 μg/mL  (i.e., 1.25 μg/mL pyrrocidine A + 1.25 μg/mL pyrrocidine B) | *F. verticillioides* WT | Δ*FvZBD1* | 19.345 | (7.578, 30.901) | 0.008 |
| Pyrrocidine A+B | 5 μg/mL  (i.e., 2.5 μg/mL pyrrocidine A + 2.5 μg/mL pyrrocidine B) | *F. verticillioides* WT | Δ*FvZBD1* | 22.104 | (12.394, 31.987) | 0.003 |
| Pyrrocidine A+B | 10 μg/mL  (i.e., 5 μg/mL pyrrocidine A + 5 μg/mL pyrrocidine B) | *F. verticillioides* WT | Δ*FvZBD1* | 39.081 | (33.458, 45.625) | < 0.001 |
| Pyrrocidine A+B | 20 μg/mL  (i.e., 10 μg/mL pyrrocidine A + 10 μg/mL pyrrocidine B) | *F. verticillioides* WT | Δ*FvZBD1* | 0.639 | (-1.018, 2.209) | 0.634* |

**Supplementary Table 9.** **The *F. verticillioides* Δ*FvZBD1* mutant exhibited extreme sensitivity to pyrrocidine A and elevated sensitivity to pyrrocidine B as demonstrated by growth rate reduction.** Comparisons of fitted model values by strain within concentration and compound, of the maximum growth rate of *F. verticillioides* WT and Δ*FvZBD1* mutant challenged with pyrrocidine A, B, or combination A+B at the following final concentrations: (1) 0.5, (2) 1.0, (3) 2.5, (4) 5.0, (5) 10, and (6) 20 μg/mL. As shown in narrower 95% credible intervals and <0.05 p_MAP_ values, Δ*FvZBD1* exhibited a significantly lower maximum growth rate under 5 μg/mL pyrrocidine A and B challenge and higher, and 2.5 μg/mL pyrrocidine A+B combined challenge and higher.

| Compound | Concentration | Strain 1 | Strain 2 | Difference Between Maximum Growth Rates | 95% Credible Interval | p_MAP_ |
| --- | --- | --- | --- | --- | --- | --- |
| Pyrrocidine A | 0.5 μg/mL | *F. verticillioides* WT | Δ*FvZBD1* | −0.003 | (-0.006, 0) | 0.176 |
| Pyrrocidine A | 1 μg/mL | *F. verticillioides* WT | Δ*FvZBD1* | −0.003 | (-0.006, 0) | 0.208 |
| Pyrrocidine A | 2.5 μg/mL | *F. verticillioides* WT | Δ*FvZBD1* | 0.002 | (-0.001, 0.005) | 0.552 |
| Pyrrocidine A | 5 μg/mL | *F. verticillioides* WT | Δ*FvZBD1* | 0.015 | (0.012, 0.017) | < 0.001 |
| Pyrrocidine A | 10 μg/mL | *F. verticillioides* WT | Δ*FvZBD1* | 0.039 | (0.036, 0.042) | < 0.001 |
| Pyrrocidine A | 20 μg/mL | *F. verticillioides* WT | Δ*FvZBD1* | 0.025 | (0.022, 0.028) | < 0.001 |
| Pyrrocidine B | 0.5 μg/mL | *F. verticillioides* WT | Δ*FvZBD1* | −0.005 | (-0.008, -0.002) | 0.003 |
| Pyrrocidine B | 1 μg/mL | *F. verticillioides* WT | Δ*FvZBD1* | −0.002 | (-0.005, 0.001) | 0.541 |
| Pyrrocidine B | 2.5 μg/mL | *F. verticillioides* WT | Δ*FvZBD1* | 0.002 | (-0.001, 0.005) | 0.447 |
| Pyrrocidine B | 5 μg/mL | *F. verticillioides* WT | Δ*FvZBD1* | 0.007 | (0.004, 0.009) | < 0.001 |
| Pyrrocidine B | 10 μg/mL | *F. verticillioides* WT | Δ*FvZBD1* | 0.014 | (0.012, 0.017) | < 0.001 |
| Pyrrocidine B | 20 μg/mL | *F. verticillioides* WT | Δ*FvZBD1* | 0.023 | (0.02, 0.026) | < 0.001 |
| Pyrrocidine A+B | 0.5 μg/mL  (i.e., 0.25 μg/mL pyrrocidine A + 0.25 μg/mL pyrrocidine B) | *F. verticillioides* WT | Δ*FvZBD1* | −0.002 | (-0.005, 0.001) | 0.378 |
| Pyrrocidine A+B | 1 μg/mL  (i.e., 0.5 μg/mL pyrrocidine A + 0.5 μg/mL pyrrocidine B) | *F. verticillioides* WT | Δ*FvZBD1* | −0.001 | (-0.004, 0.002) | 0.791 |
| Pyrrocidine A+B | 2.5 μg/mL  (i.e., 1.25 μg/mL pyrrocidine A + 1.25 μg/mL pyrrocidine B) | *F. verticillioides* WT | Δ*FvZBD1* | 0.006 | (0.003, 0.009) | < 0.001 |
| Pyrrocidine A+B | 5 μg/mL  (i.e., 2.5 μg/mL pyrrocidine A + 2.5 μg/mL pyrrocidine B) | *F. verticillioides* WT | Δ*FvZBD1* | 0.018 | (0.015, 0.02) | < 0.001 |
| Pyrrocidine A+B | 10 μg/mL  (i.e., 5 μg/mL pyrrocidine A + 5 μg/mL pyrrocidine B) | *F. verticillioides* WT | Δ*FvZBD1* | 0.023 | (0.02, 0.025) | < 0.001 |
| Pyrrocidine A+B | 20 μg/mL  (i.e., 10 μg/mL pyrrocidine A + 10 μg/mL pyrrocidine B) | *F. verticillioides* WT | Δ*FvZBD1* | 0.004 | (0.002, 0.006) | 0.005 |

**Supplementary Table 10.** **The *F. verticillioides* Δ*FvZBD1* mutant exhibited extreme sensitivity to pyrrocidine A and elevated sensitivity to pyrrocidine B as demonstrated by final OD value reduction.** Comparisons of fitted model values by strain within concentration and compound, of the optical density (OD) at the final time point of *F. verticillioides* WT and Δ*FvZBD1* mutant challenged with pyrrocidine A, B, or combination A+B at the following final concentrations: (1) 0.5, (2) 1.0, (3) 2.5, (4) 5.0, (5) 10, and (6) 20 μg/mL. As shown in narrower 95% credible intervals and <0.05 p_MAP_ values, Δ*FvZBD1* exhibited a significantly lower final OD value under 2.5 μg/mL pyrrocidine A challenge and higher, 20 μg/mL pyrrocidine B challenge, and 2.5 μg/mL pyrrocidine A+B combined challenge and higher. *To note, the 20 μg/mL pyrrocidine A+B treatment is not statistically meaningful because growth was completely inhibited for both wild type and Δ*FvZBD1* under this treatment condition.

| Compound | Concentration | Strain 1 | Strain 2 | Difference Between Final OD | 95% Credible Interval | p_MAP_ |
| --- | --- | --- | --- | --- | --- | --- |
| Pyrrocidine A | 0.5 μg/mL | *F. verticillioides* WT | Δ*FvZBD1* | 0.231 | (-0.12, 0.56) | 0.356 |
| Pyrrocidine A | 1 μg/mL | *F. verticillioides* WT | Δ*FvZBD1* | 0.324 | (-0.024, 0.645) | 0.138 |
| Pyrrocidine A | 2.5 μg/mL | *F. verticillioides* WT | Δ*FvZBD1* | 0.565 | (0.277, 0.87) | 0.003 |
| Pyrrocidine A | 5 μg/mL | *F. verticillioides* WT | Δ*FvZBD1* | 0.526 | (0.282, 0.777) | 0.003 |
| Pyrrocidine A | 10 μg/mL | *F. verticillioides* WT | Δ*FvZBD1* | 0.870 | (0.755, 1.009) | < 0.001 |
| Pyrrocidine A | 20 μg/mL | *F. verticillioides* WT | Δ*FvZBD1* | 0.551 | (0.465, 0.648) | < 0.001 |
| Pyrrocidine B | 0.5 μg/mL | *F. verticillioides* WT | Δ*FvZBD1* | 0.068 | (-0.301, 0.411) | 0.898 |
| Pyrrocidine B | 1 μg/mL | *F. verticillioides* WT | Δ*FvZBD1* | 0.138 | (-0.23, 0.479) | 0.682 |
| Pyrrocidine B | 2.5 μg/mL | *F. verticillioides* WT | Δ*FvZBD1* | 0.190 | (-0.153, 0.528) | 0.479 |
| Pyrrocidine B | 5 μg/mL | *F. verticillioides* WT | Δ*FvZBD1* | 0.303 | (0.011, 0.595) | 0.110 |
| Pyrrocidine B | 10 μg/mL | *F. verticillioides* WT | Δ*FvZBD1* | 0.310 | (0.042, 0.572) | 0.056 |
| Pyrrocidine B | 20 μg/mL | *F. verticillioides* WT | Δ*FvZBD1* | 0.492 | (0.298, 0.697) | 0.001 |
| Pyrrocidine A+B | 0.5 μg/mL  (i.e., 0.25 μg/mL pyrrocidine A + 0.25 μg/mL pyrrocidine B) | *F. verticillioides* WT | Δ*FvZBD1* | 0.217 | (-0.128, 0.563) | 0.375 |
| Pyrrocidine A+B | 1 μg/mL  (i.e., 0.5 μg/mL pyrrocidine A + 0.5 μg/mL pyrrocidine B) | *F. verticillioides* WT | Δ*FvZBD1* | 0.298 | (-0.04, 0.62) | 0.176 |
| Pyrrocidine A+B | 2.5 μg/mL  (i.e., 1.25 μg/mL pyrrocidine A + 1.25 μg/mL pyrrocidine B) | *F. verticillioides* WT | Δ*FvZBD1* | 0.534 | (0.248, 0.824) | 0.007 |
| Pyrrocidine A+B | 5 μg/mL  (i.e., 2.5 μg/mL pyrrocidine A + 2.5 μg/mL pyrrocidine B) | *F. verticillioides* WT | Δ*FvZBD1* | 0.426 | (0.177, 0.674) | 0.007 |
| Pyrrocidine A+B | 10 μg/mL  (i.e., 5 μg/mL pyrrocidine A + 5 μg/mL pyrrocidine B) | *F. verticillioides* WT | Δ*FvZBD1* | 1.005 | (0.864, 1.17) | < 0.001 |
| Pyrrocidine A+B | 20 μg/mL  (i.e., 10 μg/mL pyrrocidine A + 10 μg/mL pyrrocidine B) | *F. verticillioides* WT | Δ*FvZBD1* | 0.013 | (-0.001, 0.028) | 0.146* |

**Supplementary Table 11. The *F. verticillioides* Δ*FvABC3* mutant exhibited wild type-like growth under pyrrocidine A challenge, yet had elevated sensitivity to pyrrocidine B, as demonstrated through total growth.** Comparisons of fitted model values by strain within concentration and compound, of the total growth (area under the optical density (OD) growth curve) of *F. verticillioides* WT and Δ*FvABC3* mutant challenged with pyrrocidine A, B, or combination A+B at the following final concentrations: (1) 0.5, (2) 1.0, (3) 2.5, (4) 5.0, (5) 10, and (6) 20 μg/mL. As demonstrated in larger 95% credible intervals and >0.05 p_MAP_ values, Δ*FvABC3* exhibited wild type-like total growth under all pyrrocidine A treatments (excluding 10 μg/mL). As shown in narrower 95% credible intervals and <0.05 p_MAP_ values, Δ*FvABC3* exhibited significantly less total growth under 10 μg/mL pyrrocidine B challenge and higher, and 10 μg/mL pyrrocidine A+B combined challenge. See **Supplementary Tables 12** and **13** for comparisons of the maximum growth rate and final OD value. *To note, the 20 μg/mL pyrrocidine A+B treatment is not statistically meaningful because growth was completely inhibited for both wild type and Δ*FvABC3* under this treatment condition.

| Compound | Concentration | Strain 1 | Strain 2 | Difference Between Total Growth | 95% Credible Interval | p_MAP_ |
| --- | --- | --- | --- | --- | --- | --- |
| Pyrrocidine A | 0.5 μg/mL | *F. verticillioides* WT | Δ*FvABC3* | 3.148 | (-11.1, 17.724) | 0.894 |
| Pyrrocidine A | 1 μg/mL | *F. verticillioides* WT | Δ*FvABC3* | 2.554 | (-11.363, 17.028) | 0.923 |
| Pyrrocidine A | 2.5 μg/mL | *F. verticillioides* WT | Δ*FvABC3* | 6.466 | (-6.461, 19.984) | 0.549 |
| Pyrrocidine A | 5 μg/mL | *F. verticillioides* WT | Δ*FvABC3* | 8.393 | (-3.066, 19.798) | 0.266 |
| Pyrrocidine A | 10 μg/mL | *F. verticillioides* WT | Δ*FvABC3* | 11.075 | (5.02, 17.563) | 0.003 |
| Pyrrocidine A | 20 μg/mL | *F. verticillioides* WT | Δ*FvABC3* | 2.605 | (-2.299, 7.832) | 0.478 |
| Pyrrocidine B | 0.5 μg/mL | *F. verticillioides* WT | Δ*FvABC3* | 3.740 | (-9.612, 17.758) | 0.828 |
| Pyrrocidine B | 1 μg/mL | *F. verticillioides* WT | Δ*FvABC3* | 6.858 | (-7.482, 21.364) | 0.521 |
| Pyrrocidine B | 2.5 μg/mL | *F. verticillioides* WT | Δ*FvABC3* | 10.705 | (-3.291, 25.239) | 0.262 |
| Pyrrocidine B | 5 μg/mL | *F. verticillioides* WT | Δ*FvABC3* | 11.591 | (-1.137, 24.498) | 0.154 |
| Pyrrocidine B | 10 μg/mL | *F. verticillioides* WT | Δ*FvABC3* | 24.072 | (12.79, 35.142) | < 0.001 |
| Pyrrocidine B | 20 μg/mL | *F. verticillioides* WT | Δ*FvABC3* | 52.551 | (45.524, 60.846) | < 0.001 |
| Pyrrocidine A+B | 0.5 μg/mL  (i.e., 0.25 μg/mL pyrrocidine A + 0.25 μg/mL pyrrocidine B) | *F. verticillioides* WT | Δ*FvABC3* | 6.714 | (-7.068, 20.92) | 0.573 |
| Pyrrocidine A+B | 1 μg/mL  (i.e., 0.5 μg/mL pyrrocidine A + 0.5 μg/mL pyrrocidine B) | *F. verticillioides* WT | Δ*FvABC3* | 4.304 | (-9.69, 18.721) | 0.770 |
| Pyrrocidine A+B | 2.5 μg/mL  (i.e., 1.25 μg/mL pyrrocidine A + 1.25 μg/mL pyrrocidine B) | *F. verticillioides* WT | Δ*FvABC3* | 6.960 | (-5.933, 20.072) | 0.505 |
| Pyrrocidine A+B | 5 μg/mL  (i.e., 2.5 μg/mL pyrrocidine A + 2.5 μg/mL pyrrocidine B) | *F. verticillioides* WT | Δ*FvABC3* | 12.226 | (1.633, 23.359) | 0.070 |
| Pyrrocidine A+B | 10 μg/mL  (i.e., 5 μg/mL pyrrocidine A + 5 μg/mL pyrrocidine B) | *F. verticillioides* WT | Δ*FvABC3* | 23.769 | (17.054, 31.162) | < 0.001 |
| Pyrrocidine A+B | 20 μg/mL  (i.e., 10 μg/mL pyrrocidine A + 10 μg/mL pyrrocidine B) | *F. verticillioides* WT | Δ*FvABC3* | 0.277 | (-1.295, 1.914) | 0.914* |

**Supplementary Table 12.** **The *F. verticillioides* Δ*FvABC3* mutant exhibited wild type-like growth under pyrrocidine A challenge, yet had elevated sensitivity to pyrrocidine B, as demonstrated through growth rate.** Comparisons of fitted model values by strain within concentration and compound, of the maximum growth rate of *F. verticillioides* WT and Δ*FvABC3* mutant challenged with pyrrocidine A, B, or combination A+B at the following final concentrations: (1) 0.5, (2) 1.0, (3) 2.5, (4) 5.0, (5) 10, and (6) 20 μg/mL. As demonstrated in larger 95% credible intervals and >0.05 p_MAP_ values, Δ*FvABC3* exhibited wild type-like maximum growth rates under all pyrrocidine A treatments (excluding 10 μg/mL). As shown in narrower 95% credible intervals and <0.05 p_MAP_ values, Δ*FvABC3* exhibited a significantly lower maximum growth rate under 2.5 μg/mL pyrrocidine B challenge and higher, and 5 μg/mL pyrrocidine A+B combined challenge and higher.

| Compound | Concentration | Strain 1 | Strain 2 | Difference Between Maximum Growth Rates | 95% Credible Interval | p_MAP_ |
| --- | --- | --- | --- | --- | --- | --- |
| Pyrrocidine A | 0.5 μg/mL | *F. verticillioides* WT | Δ*FvABC3* | −0.001 | (-0.004, 0.002) | 0.878 |
| Pyrrocidine A | 1 μg/mL | *F. verticillioides* WT | Δ*FvABC3* | 0.000 | (-0.004, 0.003) | 0.985 |
| Pyrrocidine A | 2.5 μg/mL | *F. verticillioides* WT | Δ*FvABC3* | 0.000 | (-0.003, 0.003) | 0.999 |
| Pyrrocidine A | 5 μg/mL | *F. verticillioides* WT | Δ*FvABC3* | 0.003 | (0.001, 0.006) | 0.064 |
| Pyrrocidine A | 10 μg/mL | *F. verticillioides* WT | Δ*FvABC3* | 0.004 | (0.001, 0.007) | 0.012 |
| Pyrrocidine A | 20 μg/mL | *F. verticillioides* WT | Δ*FvABC3* | −0.003 | (-0.006, 0) | 0.181 |
| Pyrrocidine B | 0.5 μg/mL | *F. verticillioides* WT | Δ*FvABC3* | 0.001 | (-0.002, 0.003) | 0.923 |
| Pyrrocidine B | 1 μg/mL | *F. verticillioides* WT | Δ*FvABC3* | 0.001 | (-0.002, 0.004) | 0.707 |
| Pyrrocidine B | 2.5 μg/mL | *F. verticillioides* WT | Δ*FvABC3* | 0.004 | (0.002, 0.007) | 0.011 |
| Pyrrocidine B | 5 μg/mL | *F. verticillioides* WT | Δ*FvABC3* | 0.006 | (0.004, 0.009) | < 0.001 |
| Pyrrocidine B | 10 μg/mL | *F. verticillioides* WT | Δ*FvABC3* | 0.016 | (0.013, 0.019) | < 0.001 |
| Pyrrocidine B | 20 μg/mL | *F. verticillioides* WT | Δ*FvABC3* | 0.067 | (0.065, 0.07) | < 0.001 |
| Pyrrocidine A+B | 0.5 μg/mL  (i.e., 0.25 μg/mL pyrrocidine A + 0.25 μg/mL pyrrocidine B) | *F. verticillioides* WT | Δ*FvABC3* | 0.001 | (-0.002, 0.004) | 0.708 |
| Pyrrocidine A+B | 1 μg/mL  (i.e., 0.5 μg/mL pyrrocidine A + 0.5 μg/mL pyrrocidine B) | *F. verticillioides* WT | Δ*FvABC3* | 0.000 | (-0.003, 0.003) | 0.998 |
| Pyrrocidine A+B | 2.5 μg/mL  (i.e., 1.25 μg/mL pyrrocidine A + 1.25 μg/mL pyrrocidine B) | *F. verticillioides* WT | Δ*FvABC3* | 0.002 | (-0.001, 0.004) | 0.539 |
| Pyrrocidine A+B | 5 μg/mL  (i.e., 2.5 μg/mL pyrrocidine A + 2.5 μg/mL pyrrocidine B) | *F. verticillioides* WT | Δ*FvABC3* | 0.008 | (0.005, 0.01) | < 0.001 |
| Pyrrocidine A+B | 10 μg/mL  (i.e., 5 μg/mL pyrrocidine A + 5 μg/mL pyrrocidine B) | *F. verticillioides* WT | Δ*FvABC3* | 0.013 | (0.011, 0.015) | < 0.001 |
| Pyrrocidine A+B | 20 μg/mL  (i.e., 10 μg/mL pyrrocidine A + 10 μg/mL pyrrocidine B) | *F. verticillioides* WT | Δ*FvABC3* | 0.004 | (0.002, 0.006) | 0.003 |

**Supplementary Table 13.** **The *F. verticillioides* Δ*FvABC3* mutant exhibited wild type-like growth under pyrrocidine A challenge, yet had elevated sensitivity to pyrrocidine B, as demonstrated through the final OD value.** Comparisons of fitted model values by strain within concentration and compound, of the optical density (OD) at the final time point of *F. verticillioides* WT and Δ*FvABC3* mutant challenged with pyrrocidine A, B, or combination A+B at the following final concentrations: (1) 0.5, (2) 1.0, (3) 2.5, (4) 5.0, (5) 10, and (6) 20 μg/mL. As demonstrated in larger 95% credible intervals and >0.05 p_MAP_ values, Δ*FvABC3* exhibited wild type-like final OD values under all pyrrocidine A treatments. As shown in narrower 95% credible intervals and <0.05 p_MAP_ values, Δ*FvABC3* exhibited a significantly lower final OD value under 10 μg/mL pyrrocidine B challenge and higher, and 10 μg/mL pyrrocidine A+B combined challenge. *To note, the 20 μg/mL pyrrocidine A+B treatment is not statistically meaningful because growth was completely inhibited for both wild type and Δ*FvABC3* under this treatment condition.

| Compound | Concentration | Strain 1 | Strain 2 | Difference Between Final OD | 95% Credible Interval | p_MAP_ |
| --- | --- | --- | --- | --- | --- | --- |
| Pyrrocidine A | 0.5 μg/mL | *F. verticillioides* WT | Δ*FvABC3* | 0.072 | (-0.285, 0.432) | 0.914 |
| Pyrrocidine A | 1 μg/mL | *F. verticillioides* WT | Δ*FvABC3* | 0.066 | (-0.285, 0.427) | 0.918 |
| Pyrrocidine A | 2.5 μg/mL | *F. verticillioides* WT | Δ*FvABC3* | 0.155 | (-0.181, 0.503) | 0.556 |
| Pyrrocidine A | 5 μg/mL | *F. verticillioides* WT | Δ*FvABC3* | 0.199 | (-0.08, 0.482) | 0.324 |
| Pyrrocidine A | 10 μg/mL | *F. verticillioides* WT | Δ*FvABC3* | 0.136 | (-0.037, 0.32) | 0.263 |
| Pyrrocidine A | 20 μg/mL | *F. verticillioides* WT | Δ*FvABC3* | 0.041 | (-0.088, 0.176) | 0.762 |
| Pyrrocidine B | 0.5 μg/mL | *F. verticillioides* WT | Δ*FvABC3* | 0.074 | (-0.273, 0.43) | 0.893 |
| Pyrrocidine B | 1 μg/mL | *F. verticillioides* WT | Δ*FvABC3* | 0.101 | (-0.256, 0.459) | 0.837 |
| Pyrrocidine B | 2.5 μg/mL | *F. verticillioides* WT | Δ*FvABC3* | 0.204 | (-0.137, 0.552) | 0.419 |
| Pyrrocidine B | 5 μg/mL | *F. verticillioides* WT | Δ*FvABC3* | 0.235 | (-0.056, 0.539) | 0.242 |
| Pyrrocidine B | 10 μg/mL | *F. verticillioides* WT | Δ*FvABC3* | 0.445 | (0.188, 0.703) | 0.002 |
| Pyrrocidine B | 20 μg/mL | *F. verticillioides* WT | Δ*FvABC3* | 1.192 | (1.039, 1.371) | < 0.001 |
| Pyrrocidine A+B | 0.5 μg/mL  (i.e., 0.25 μg/mL pyrrocidine A + 0.25 μg/mL pyrrocidine B) | *F. verticillioides* WT | Δ*FvABC3* | 0.119 | (-0.229, 0.483) | 0.743 |
| Pyrrocidine A+B | 1 μg/mL  (i.e., 0.5 μg/mL pyrrocidine A + 0.5 μg/mL pyrrocidine B) | *F. verticillioides* WT | Δ*FvABC3* | 0.089 | (-0.267, 0.453) | 0.826 |
| Pyrrocidine A+B | 2.5 μg/mL  (i.e., 1.25 μg/mL pyrrocidine A + 1.25 μg/mL pyrrocidine B) | *F. verticillioides* WT | Δ*FvABC3* | 0.177 | (-0.146, 0.505) | 0.503 |
| Pyrrocidine A+B | 5 μg/mL  (i.e., 2.5 μg/mL pyrrocidine A + 2.5 μg/mL pyrrocidine B) | *F. verticillioides* WT | Δ*FvABC3* | 0.269 | (0.005, 0.538) | 0.117 |
| Pyrrocidine A+B | 10 μg/mL  (i.e., 5 μg/mL pyrrocidine A + 5 μg/mL pyrrocidine B) | *F. verticillioides* WT | Δ*FvABC3* | 0.693 | (0.537, 0.869) | < 0.001 |
| Pyrrocidine A+B | 20 μg/mL  (i.e., 10 μg/mL pyrrocidine A + 10 μg/mL pyrrocidine B) | *F. verticillioides* WT | Δ*FvABC3* | 0.011 | (-0.003, 0.026) | 0.290* |

**Supplementary Table 14. 0.5% DMSO does not significantly affect the *F. verticillioides* final OD value.** Comparisons of fitted model values of the optical density (OD) at the final time point of the *F. verticillioides* WT, Δ*FvZBD1*, and Δ*FvABC3* no-treatment control and 0.5% DMSO control, within each *F. verticillioides* strain. Based on the large 95% credible intervals and >0.05 p_MAP_ values, there was no meaningful statistical difference in the final OD value between the no-treatment control and DMSO control for the *F. verticillioides* WT, Δ*FvZBD1*, or Δ*FvABC3* strains.

| Strain | Difference Between Final OD in No-Treatment Control and 0.5% DMSO Control | 95% Credible Interval | p_MAP_ |
| --- | --- | --- | --- |
| *F. verticillioides* WT | 0.033 | (-0.125, 0.19) | 0.932 |
| Δ*FvZBD1* | −0.022 | (-0.195, 0.154) | 0.973 |
| Δ*FvABC3* | 0.012 | (-0.167, 0.193) | 0.996 |

**Supplementary Table 15. 0.5% DMSO does not affect *F. verticillioides* maximum growth rate in a biologically relevant manner.** Comparisons of fitted model values of the maximum growth rate of the *F. verticillioides* WT, Δ*FvZBD1*, and Δ*FvABC3* no-treatment control and 0.5% DMSO control, within each *F. verticillioides* strain. Based on the large 95% credible intervals and >0.05 p_MAP_ values, there was no meaningful statistical difference in the maximum growth rate between the no-treatment control and DMSO control for the *F. verticillioides* Δ*FvZBD1* or Δ*FvABC3* strains. In *F. verticillioides* WT, the DMSO maximum growth rate was slightly less than that of the no-treatment control (p_MAP_ = 0.021).

| Strain | Difference Between Maximum Growth Rates in No-Treatment Control and 0.5% DMSO Control | 95% Credible Interval | p_MAP_ |
| --- | --- | --- | --- |
| *F. verticillioides* WT | 0.004 | (0.001, 0.007) | 0.021 |
| Δ*FvZBD1* | 0.001 | (-0.002, 0.004) | 0.805 |
| Δ*FvABC3* | 0.002 | (-0.001, 0.005) | 0.597 |
